# Autophosphorylation at serine 166 regulates RIP kinase 1-mediated cell death and inflammation

**DOI:** 10.1038/s41467-020-15466-8

**Published:** 2020-04-08

**Authors:** Lucie Laurien, Masahiro Nagata, Hannah Schünke, Tom Delanghe, Janica L. Wiederstein, Snehlata Kumari, Robin Schwarzer, Teresa Corona, Marcus Krüger, Mathieu J. M. Bertrand, Vangelis Kondylis, Manolis Pasparakis

**Affiliations:** 1 Institute for Genetics, Cologne Excellence Cluster on Cellular Stress Responses in Aging-Associated Diseases (CECAD), University of Cologne, Cologne, Germany; 2 VIB Center for Inflammation Research, Technologiepark-Zwinjaarde 71, 9052 Zwinjaarde-Ghent, Belgium; 3 Department of Biomedical Molecular Biology, Ghent University, Technologiepark-Zwinjaarde 71, 9052 Zwinaarde-Ghent, Belgium; 4 Center for Molecular Medicine Cologne, University of Cologne, Cologne, Germany

**Keywords:** Apoptosis, Necroptosis

## Abstract

Receptor interacting protein kinase 1 (RIPK1) regulates cell death and inflammatory responses downstream of TNFR1 and other receptors, and has been implicated in the pathogenesis of inflammatory and degenerative diseases. RIPK1 kinase activity induces apoptosis and necroptosis, however the mechanisms and phosphorylation events regulating RIPK1-dependent cell death signaling remain poorly understood. Here we show that RIPK1 autophosphorylation at serine 166 plays a critical role for the activation of RIPK1 kinase-dependent apoptosis and necroptosis. Moreover, we show that S166 phosphorylation is required for RIPK1 kinase-dependent pathogenesis of inflammatory pathologies in vivo in four relevant mouse models. Mechanistically, we provide evidence that trans autophosphorylation at S166 modulates RIPK1 kinase activation but is not by itself sufficient to induce cell death. These results show that S166 autophosphorylation licenses RIPK1 kinase activity to induce downstream cell death signaling and inflammation, suggesting that S166 phosphorylation can serve as a reliable biomarker for RIPK1 kinase-dependent pathologies.

## Abstract (web summary)

Receptor interacting protein kinase 1 (RIPK1) regulates cell death and inflammatory responses. Here the authors show that autophosphorylation at Ser166 is required for RIPK1-mediated cell death and inflammation in mouse models of inflammatory pathologies, making Ser166 phosphorylation a possible biomarker for RIPK1-mediated inflammatory diseases.

## Introduction

Receptor interacting protein kinase 1 (RIPK1) has emerged as a key player regulating cell death and inflammation, which is implicated in the pathogenesis of inflammatory and degenerative diseases^1,2^. RIPK1 regulates signaling downstream of tumor necrosis factor receptor 1 (TNFR1) as well as toll like receptors (TLR) 3 and 4, by exhibiting both kinase activity-dependent and -independent functions. Studies in RIPK1-deficient mice and cells showed that RIPK1 acts as a scaffold independently of its kinase activity to regulate pro-inflammatory and pro-survival TNFR1 and TLR3/4 signaling, a function that is essential for mouse development and tissue homeostasis^3–10^. In contrast, RIPK1 kinase activity induces cell death by activating either caspase-8-dependent apoptosis or RIPK3-mixed lineage kinase like (MLKL)-dependent necroptosis^1,2,11,12^. Multiple studies using genetic or pharmacological inhibition have identified RIPK1 kinase activity-dependent cell death as a potent trigger of inflammation in different tissues^13–17^. Furthermore, RIPK1 kinase activity emerged as driver of ischemic injury^18–20^ as well as neurodegenerative diseases such as multiple sclerosis (MS)^21^, ALS (amyotrophic lateral sclerosis)^22^ and Alzheimer’s disease^23^. These studies identified RIPK1 kinase activity as a key factor contributing to the pathogenesis of inflammatory diseases, prompting the development of RIPK1 kinase inhibitors^24–28^ that reached clinical trials for the treatment of inflammatory and neurodegenerative diseases as well as pancreatic cancer^24,25^.

The critical role of RIPK1 in driving cell death and inflammation motivated several studies addressing the mechanisms regulating its cytotoxic and pathogenic potential. Recent studies reported that RIPK1 phosphorylation by a number of kinases, including inhibitor of NF-κB (IκB) kinases (IKKs), mitogen activated protein kinase (MAPK)-activated protein kinase 2 (MK2), transforming growth factor beta activating kinase 1 (TAK1), as well as TANK-binding kinase 1 (TBK1) and IKKε, limits TNF-induced RIPK1 kinase-dependent cell death and inflammation in vitro and in vivo^22,29–35^. These results revealed that RIPK1 kinase activity is tightly controlled by a multitude of mechanisms, consistent with its strong cytotoxic potential.

Despite the well-established role of RIPK1 kinase activity in driving cell death and inflammatory pathologies, it remains unclear how RIPK1 kinase activity regulates downstream signaling. Although RIPK1 was also proposed to phosphorylate other substrates such as DRP1 (ref. ^36^), autophosphorylation is currently considered the main and critical function of RIPK1 kinase activity. Several autophosphorylation sites have been detected in mouse RIPK1, including serine residues 14/15, 161 and 166, as well as threonine 169 (refs. ^13,32,37,38^), while autophosphorylation at serines 14/15, 20, 161 and 166 was reported for human RIPK1 (ref. ^37^). The currently prevailing model is that autophosphorylation induces conformational changes in RIPK1 that allow its association with cell death effectors including fas-associated with death domain (FADD) and RIPK3, facilitating the formation of cell death-inducing signaling complexes causing apoptosis or necroptosis^39^. However, the role of specific autophosphorylation events and the mechanisms by which these trigger cell death remain poorly understood. Using in vitro cellular systems, two independent studies reported that alanine substitution at S161 leads to a reduction in RIPK1 kinase activity^37,40^. More recently, phosphorylation at S161 was proposed to promote an “open conformation” of the T-loop, thereby facilitating RIPK1-RIPK3 interaction to promote cell death^38^, suggesting a major functional importance of S161. Apart from S161, none of the other residues seemed to have a major effect on cell death induction^40^. While RIPK1 autophosphorylation at S166 is commonly employed as a biomarker of RIPK1 kinase activity^10,21,35,41^, the functional role and mechanism of action of this autophosphorylation site remains elusive.

Here we identified RIPK1 autophosphorylation at S166 as a critical event for RIPK1-mediated apoptosis and necroptosis. Importantly, we show that S166 phosphorylation is essential for the pathogenesis of RIPK1-mediated inflammatory conditions in vivo in four relevant mouse models of inflammation. Mechanistically, we provide evidence that S166 phosphorylation regulates the kinase activity of RIPK1, but is not sufficient by itself to impose the conformational changes of RIPK1 that are required for the activation of downstream cell death signaling.

## Results

### S166 autophosphorylation drives RIPK1-dependent cell death

**Fig. 1:**
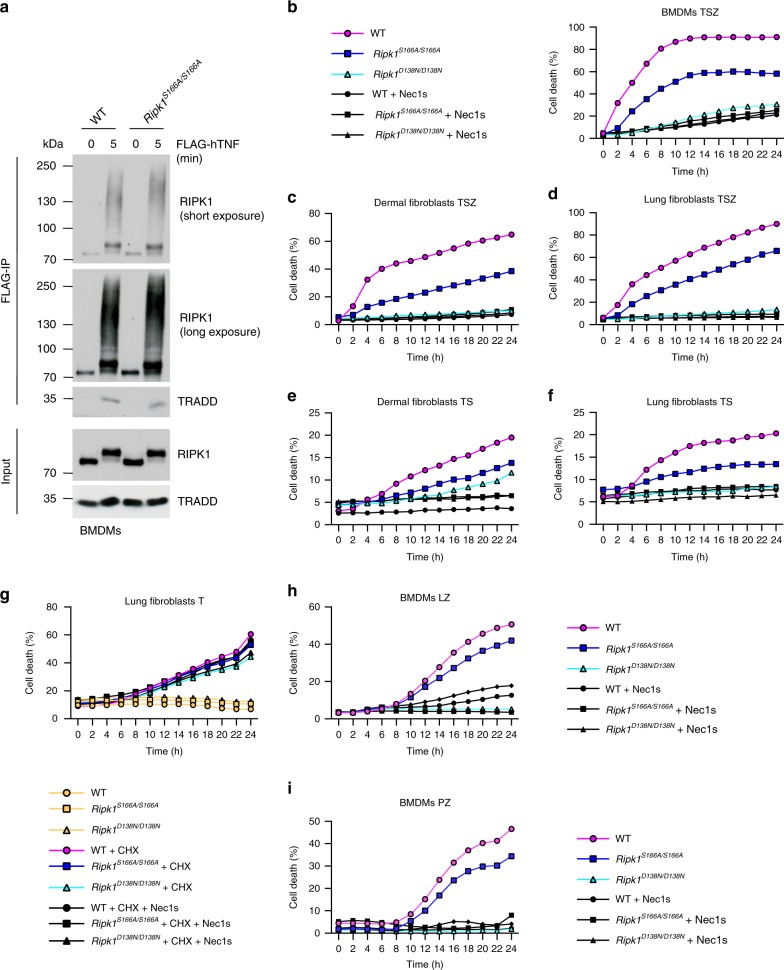
S166 phosphorylation drives RIPK1-dependent cell death. **a** BMDMs from mice of the indicated genotypes were treated with FLAG-hTNF for 0 or 5 min and FLAG-immunoprecipitates were analyzed with the indicated antibodies. Representative data of two independent experiments is shown. **b** BMDMs from mice of the indicated genotypes were treated with a combination of TNF (T; 10 ng ml^−1^), Z-VAD-FMK (Z) and SMAC mimetic (S (Birinapant); 1 µM) in the presence or absence of Nec-1s. **c** Primary dermal fibroblasts from mice of the indicated genotypes were treated with combinations of TNF (T; 20 ng ml^−1^), Z-VAD-FMK (Z) and SMAC mimetic (S (Birinapant); 1.5 µM) in the presence or absence of Nec1s. **d** Primary lung fibroblasts from mice of the indicated genotypes were treated with combinations of TNF (T; 20 ng ml^−1^), Z-VAD-FMK (Z) and SMAC mimetic (S (Birinapant); 1 µM) in the presence or absence of Nec-1s. **e** Primary dermal fibroblasts from mice of the indicated genotypes were treated with combinations of TNF (T; 20 ng ml^−1^) and SMAC mimetic (S (Birinapant); 1.5 µM) in the presence or absence of Nec-1s. **f** Primary lung fibroblasts from mice of the indicated genotypes were treated with combinations of TNF (T; 20 ng ml^−1^) and SMAC mimetic (S (Birinapant); 1 µM) in the presence or absence of Nec-1s. **g** Primary lung fibroblasts from mice of the indicated genotypes were treated with TNF (20 ng ml^−1^) in the presence or absence of cycloheximide (CHX, 1 μg ml^−1^) and Nec1-s. **h** BMDMs were treated with LPS (L; 100 ng ml^−1^) and Z-VAD-FMK (Z) in the presence or absence of Nec-1s. **i** BMDMs were treated with Poly(I:C) (P; 0.5 μg ml^−1^) and Z-VAD-FMK (Z) in the presence or absence of Nec-1s. **b**–**i** Cell death was measured using an IncuCyte as described. Graphs represent four independent experiments in **b**, **d**, **f** and **h**; three independent experiments in **c**, **e** and **i** and two independent experiments in **g**. Source data for **b**–**i** are provided as a source data file.

To address the role of RIPK1 autophosphorylation in the regulation of RIPK1-mediated cell death and inflammatory responses, we generated knock-in mice expressing RIPK1 with substitution of serine at position 166 with alanine (RIPK1S166A mutation) from the endogenous *Ripk1* genomic locus (Supplementary Fig. 1a). *Ripk1*^*S166A/S166A*^ mice were born at the expected Mendelian frequency and reached adulthood without showing signs of pathology, demonstrating that inhibition of RIPK1 phosphorylation at S166 does not interfere with normal mouse development and homeostasis under steady state conditions. Immunoblot analysis of bone marrow derived macrophages (BMDMs) from *Ripk1*^*S166A/S166A*^ mice showed that the mutated RIPK1S166A protein was expressed at similar levels as wild-type (WT) RIPK1 (Supplementary Fig. 1b, c). Moreover, *Ripk1*^*S166A/S166A*^ BMDMs showed normal activation of NF-κB and MAPK signaling in response to TNF or LPS stimulation (Supplementary Fig. 1b, c), as well as normal LPS-induced expression of NF-κB-dependent genes (Supplementary Fig. 1d). The recruitment and ubiquitination of RIPK1 within the TNFR1 signaling complex (often referred to as complex I) regulates pro-inflammatory and pro-survival signaling^42^. In order to determine whether S166A mutation affects RIPK1 recruitment and ubiquitination in complex I, we stimulated BMDMs with FLAG-tagged hTNF and immunoprecipitated the activated TNFR1 using anti-FLAG antibodies. Immunoblot analysis revealed that the S166A mutation did not affect the recruitment and ubiquitination of RIPK1 in complex I (Fig. 1a). Overall, these results suggest that the S166A mutation did not affect the scaffolding functions of RIPK1 that regulate proinflammatory signaling and tissue homeostasis.

We then asked whether autophosphorylation at S166 plays a role in RIPK1 kinase-dependent cell death downstream of TNFR1. We thus treated BMDMs as well as dermal and lung fibroblasts from WT, *Ripk1*^*S166A/S166A*^ and *Ripk1*^*D138N/D138N*^ mice, which express catalytically inactive RIPK1 (ref. ^43^), with TNF in combination with the SMAC-mimetic compound birinapant (S) and the pan-caspase inhibitor Z-VAD-FMK (Z) to induce necroptosis^13,43^. Assessment of cell death with IncuCyte real-time imaging revealed that the RIPK1S166A mutation considerably protected cells from TSZ-induced necroptosis (Fig. 1b–d). Under the same conditions, inhibition of RIPK1 kinase activity either genetically in *Ripk1*^*D138N/D138N*^ cells or pharmacologically by treatment with Necrostatin-1s (Nec1s) strongly prevented necroptosis (Fig. 1b–d). To assess whether S166 phosphorylation is also critical for TNF-induced apoptosis we treated dermal and lung fibroblasts with TNF in the presence of birinapant (TS). *Ripk1*^*S166A/S166A*^ dermal and lung fibroblasts were partially protected from TS-induced death (Fig. 1e, f), suggesting that S166 phosphorylation is important also for the induction of RIPK1-mediated apoptosis. Of note, RIPK1S166A mutation did not prevent cell death induced by TNF in the presence of cycloheximide (C) in lung fibroblasts, which was also not affected by inhibition of RIPK1 kinase activity in *Ripk1*^*D138N/D138N*^ cells or by Nec-1s treatment as previously reported^44^ (Fig. 1g). We then assessed the role of S166 phosphorylation in LPS or poly(I:C)-induced necroptosis by measuring cell death of BMDMs in response to treatment with LPS + Z-VAD-FMK (LZ) or poly(I:C) + Z-VAD-FMK (PZ), respectively. As shown in Fig. 1h, i, *Ripk1*^*S166A/S166A*^ BMDMs were partially protected from LZ- and PZ-induced necroptosis compared to WT cells, as opposed to *Ripk1*^*D138N/D138N*^ or Nec-1s-treated BMDMs that were fully protected. Taken together, our results showed that abolishing autophosphorylation at S166 did not affect RIPK1 scaffolding functions regulating the activation of pro-inflammatory and pro-survival NF-κB and MAPK signaling, but considerably inhibited RIPK1 kinase activity-dependent cell death downstream of TNFR1, TLR3 and TLR4.

### S166 phosphorylation facilitates necrosome formation

**Fig. 2:**
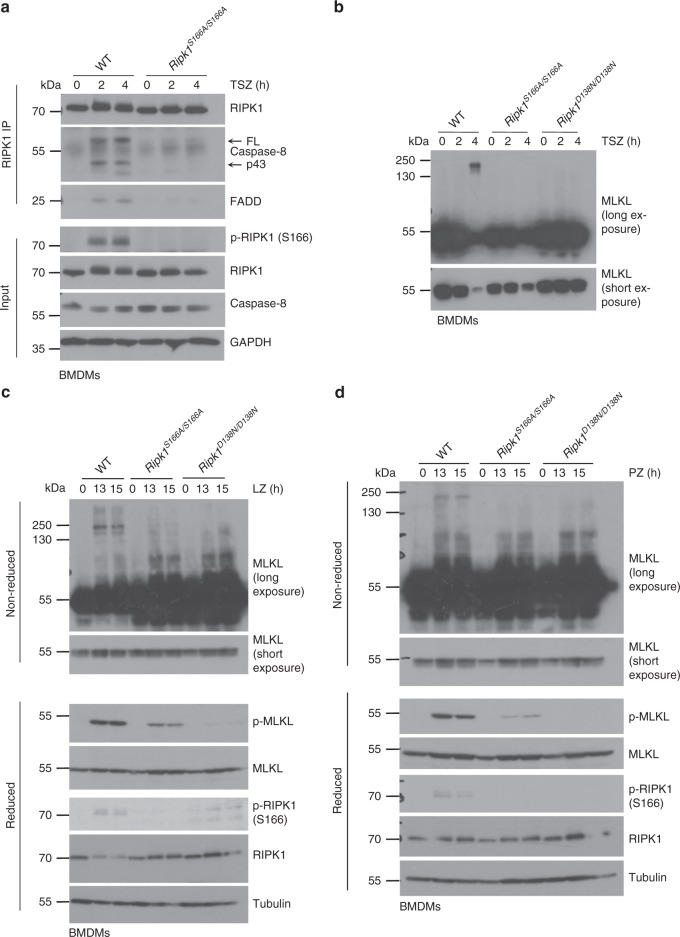
S166 phosphorylation facilitates necrosome formation. **a** BMDMs from mice of the indicated genotypes were treated with TNF (T; 10 ng ml^−1^), SMAC mimetic (S (Birinapant); 1 µM) and Z-VAD-FMK (Z) for 0, 2 and 4 h. RIPK1 immunoprecipitates and total cell lysates (input) were analyzed by immunoblotting with the indicated antibodies. **b** BMDMs from mice of the indicated genotypes were treated with TNF (T; 10 ng ml^−1^), SMAC mimetic (S (Birinapant); 1 µM) and Z-VAD-FMK (Z) for 2 and 4 h. Lysates were processed under non-reducing conditions and analyzed for presence of MLKL by immunoblot. **c** BMDMs from mice of the indicated genotypes were treated with LPS (L; 100 ng ml^−1^) and Z- VAD-FMK (Z) for 13 and 15 h. Lysates were processed under non-reducing or reducing conditions and analyzed by immunoblot using the indicated antibodies. **d** BMDMs from mice of the indicated genotypes were treated with Poly(I:C) (P; 0.5 μg ml^−1^) and Z-VAD-FMK (Z) for 13 and 15 h. Lysates were processed under non-reducing or reducing conditions and analyzed by immunoblot using the indicated antibodies. **a**, **c** Representative data of three independent experiments is shown. **b**, **d** Representative data of two independent experiments is shown.

To investigate how absence of phosphorylation at S166 impairs RIPK1 kinase activity-dependent cell death, we assessed the formation of the RIPK1-dependent necroptosis-inducing signaling complex known as complex IIb or necrosome. Therefore, we treated BMDMs with TSZ and analyzed the interaction of RIPK1 with FADD and caspase-8 by immunoprecipitating RIPK1 with specific antibodies followed by immunoblotting. In WT BMDMs, we detected strong association of RIPK1 with FADD and caspase-8 two and four hours after TSZ treatment, concurrent with RIPK1 phosphorylation at S166 and cleavage of caspase-8 (Fig. 2a). In *Ripk1*^*S166A/S166A*^ cells on the other hand, we did not observe association of RIPK1 with FADD or with caspase-8 (Fig. 2a), suggesting that loss of autophosphorylation at S166 strongly inhibited the ability of RIPK1 to nucleate the formation of the necrosome.

Necroptosis relies on MLKL phosphorylation by RIPK3 and the subsequent assembly of MLKL oligomers that translocate to the plasma membrane to execute cell lysis^45,46^. To determine whether RIPK1 autophosphorylation at S166 is required for MLKL oligomer formation upon necroptotic cell death induction, we analyzed whole cell lysates from TSZ-treated WT, *Ripk1*^*S166A/S166A*^ and *Ripk1*^*D138N/D138N*^ BMDMs under non-reducing conditions by immunoblotting with anti-MLKL antibodies. Whereas a high molecular weight MLKL band was detected in WT cells 4 h after TSZ stimulation, this shift was absent in *Ripk1*^*S166A/S166A*^ and also *Ripk1*^*D138N/D138N*^ cells (Fig. 2b). Similarly, S166A mutation strongly inhibited phosphorylation and oligomerization of MLKL in response to LZ and PZ treatment (Fig. 2c, d). Collectively, these results showed that RIPK1 autophosphorylation at S166 is critical for necrosome formation and MLKL activation in response to TSZ, LZ and PZ stimulation.

### S166A mutation prevents RIPK1-dependent colitis

**Fig. 3:**
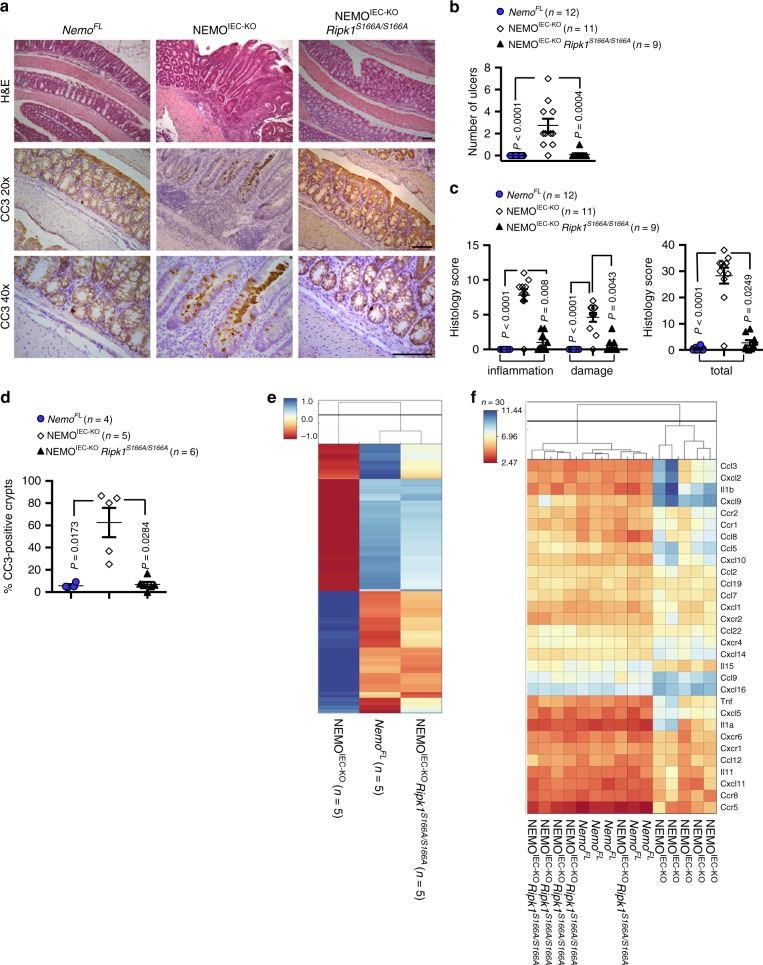
S166 phosphorylation is essential for RIPK1-dependent intestinal inflammation in NEMO^IEC-KO^ mice. **a** Representative images of colon sections from *Nemo*^*FL*^, NEMO^IEC-KO^, and NEMO^IEC-KO^ *Ripk1*^*S166A/S166A*^ littermates stained with Hematoxylin and Eosin (H&E) or immunostained for cleaved caspase-3 (CC3) at the age of 11 weeks. CC3 staining is shown in 20× and 40× magnification. Scale bars, 100 µm. **b** Graph depicting numbers of ulcers on colon sections from NEMO^IEC-KO^, *Nemo*^*FL*^ and NEMO^IEC-KO^ *Ripk1*^*S166A/S166A*^ mice. One representative out of two independend blinded countings is shown. Statistical significance was determined using a Kruskal-Wallis test (one-sided). **c** Graph depicting histology scores for inflammation, tissue damage, and overall histopathology (total) of colon sections from NEMO^IEC-KO^, *Nemo*^*FL*^ and NEMO^IEC-KO^ *Ripk1*^*S166A/S166A*^ mice. One representative out of two independent blinded scorings is shown. Statistical significance was determined using a Kruskal-Wallis test (one-sided). **d** Graph depicting percentage of crypts containing cleaved caspase-3 (CC3) positive cells in mice of the indicated genotypes. Statistical significance was determined using a Kruskal-Wallis test (one-sided). **e** Clustered heat maps showing whole genome microarray analysis of gene expression in colons from *Nemo*^*FL*^, NEMO^IEC-KO^ and NEMO^IEC-KO^ Ripk1^*S166A/S166A*^ mice. Only genes that were differentially regulated (Fold change > 1.4999 or <−1.4999; *P* < 0.05) between *Nemo*^*FL*^ and NEMO^IEC-KO^ mice were included. The values were normalized using Z score transformation. **f** Clustered heat maps showing expression of 30 cytokines that were differentially regulated (Fold change >1.4999 or <−1.4999; P < 0.05) between *Nemo*^*FL*^ and NEMO^IEC-KO^ mice. Values of individual mice are plotted; *n* = 5 per group. **b**–**d** The dots in the graphs represent individual mice. Horizontal lines indicate mean values ± s.e.m. Source data for **b**–**d** are provided as a source data file.

To investigate whether autophosphorylation at S166 regulates RIPK1-mediated cell death also in vivo, we assessed the effect of the S166A mutation in mouse models of inflammation caused by RIPK1 kinase activity-dependent cell death. Mice with intestinal-epithelial cell (IEC)-specific ablation of NEMO (NEMO^IEC-KO^) spontaneously develop severe colitis, which is caused by TNF-induced RIPK1 kinase-dependent death of IECs^15,47^. We therefore crossed NEMO^IEC-KO^ with *Ripk1*^*S166A/S166A*^ mice to investigate whether RIPK1 autophosphorylation at S166 plays a role in driving the intestinal pathology. Immunohistological analysis of colonic sections from 11-week-old mice revealed severe intestinal pathology in NEMO^IEC-KO^ mice, characterized by epithelial thickening, ulcer formation and immune cell infiltration into the colonic mucosa and submucosa, as well as an increased number of apoptotic IECs detected by immunostaining against cleaved caspase-3 (CC3) (Fig. 3a–d), as previously reported^15^. In contrast, analysis of colon sections from NEMO^IEC-KO^ *Ripk1*^*S166A/S166A*^ mice revealed a largely normal architecture without signs of IEC death and mucosal inflammation (Fig. 3a–d). Moreover, whole genome microarray gene expression analysis of colonic RNA confirmed that the *Ripk1*^*S166A/S166A*^ mutation prevented colon inflammation in NEMO^IEC-KO^ mice. Specifically, the gene expression profile of NEMO^IEC-KO^ *Ripk1*^*S166A/S166A*^ was similar to *Nemo*^*FL*^ mice as opposed to the profile of NEMO^IEC-KO^ mice, which showed altered expression of a number of genes including several inflammatory cytokines and chemokines such as *Tnf*, *Ccl5*, *Cxcl1* and *Il1b* (Fig. 3e, f). The protective effect of the S166A mutation was comparable to that offered by the kinase inactive RIPK1D138N mutation^15^, showing that autophosphorylation at S166 is essential for RIPK1 kinase activity-dependent IEC death and colitis development in NEMO^IEC-KO^ mice.

### S166A mutation prevents RIPK1-dependent hepatitis and cancer

**Fig. 4:**
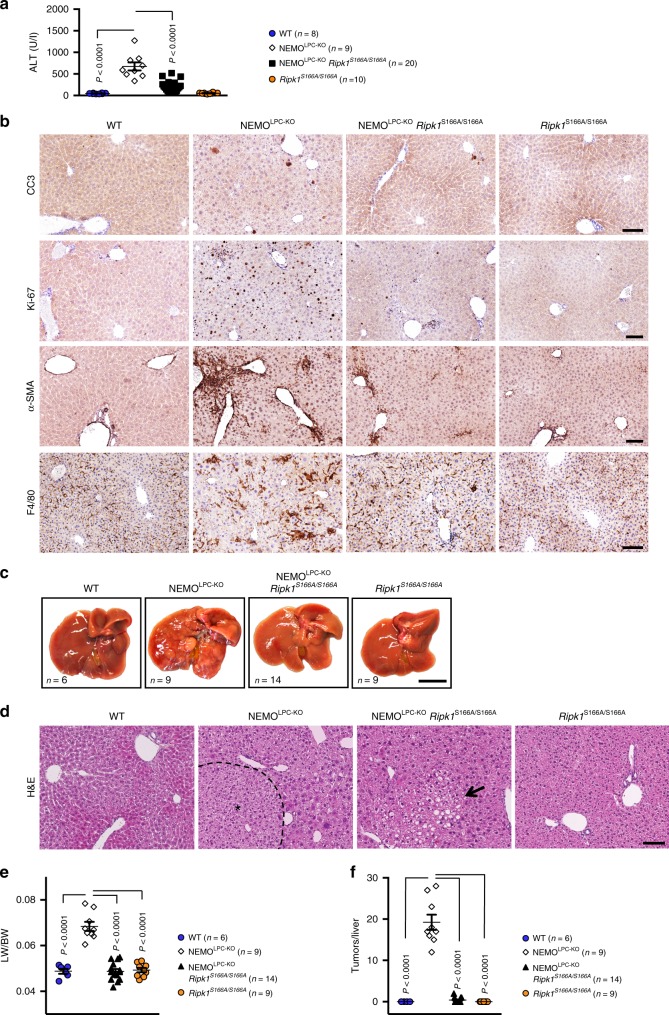
S166 phosphorylation is essential for RIPK1-dependent hepatitis and liver tumorigenesis in NEMO^LPC-KO^ mice. **a** Graph depicting basal serum alanine aminotransferase (ALT) levels in 8-week-old mice of the indicated genoytpes. **b** Representative images of liver sections from 8-week-old mice of the indicated genotypes that were stained with H&E or immunostained with the indicated antibodies. Scale bars, 200 μm. **c** Representative photographs of livers from 50-week-old mice with the indicated genotypes. Scale bar, 1 cm. **d** Representative images of liver sections from 50-week-old mice of the indicated genotypes stained with H&E. Hepatocellular carcinoma (HCC) areas are outlined and marked with an asterisk. Arrow points at a small steatotic area observed in some NEMO^LPC-KO^ *Ripk1*^*S166A/S166A*^ mice. Scale bars, 200 μm. **e** Tumor load quantification in 50-week-old mice by liver weight/body weight (LW/BW) ratios. **f** Graph depicting tumor number per liver for 50-week-old mice of the indicated genotypes. **a**, **e**, **f** The dots in the graphs represent individual mice. Horizontal lines indicate mean values ± s.e.m. Statistical significance was determined using one-way ANOVA. Source data for **a**, **e**, **f** are provided as source data file.

Mice with liver parenchymal cell (LPC)-specific NEMO deficiency (NEMO^LPC-KO^) spontaneously develop steatohepatitis and hepatocellular carcinoma (HCC), which is driven by RIPK1 kinase-dependent hepatocyte apoptosis^14,48,49^. We assessed the role of S166 phosphorylation in the development of the liver pathology by crossing the NEMO^LPC-KO^ mice with *Ripk1*^*S166A/S166A*^ animals. As shown previously^14,48,49^, NEMO^LPC-KO^ mice presented with elevated alanine aminotransferase (ALT) levels in the serum at the age of 8 weeks, indicative for liver damage (Fig. 4a). In contrast, NEMO^LPC-KO^ *Ripk1*^*S166A/S166A*^ mice showed strongly reduced serum ALT levels at this age, indicating that abolishing autophosphorylation at S166 prevented liver damage in NEMO^LPC-KO^ mice similarly to the lack of RIPK1 kinase activity^14^. Immunohistochemical analysis of liver sections from 8-week-old NEMO^LPC-KO^ *Ripk1*^*S166A/S166A*^ mice revealed that loss of S166 phosphorylation strongly prevented apoptosis and compensatory proliferation of hepatocytes, hepatic stellate cell activation and macrophage infiltration (Fig. 4b), consistent with the reduced serum ALT levels in these mice. Furthermore, analysis of livers from 50-week-old mice showed that the RIPK1S166A mutation prevented liver tumor development in NEMO^LPC-KO^ mice (Fig. 4c–f). Thus, RIPK1 autophosphorylation at S166 is essential for RIPK1 kinase activity-dependent hepatocyte apoptosis, chronic hepatitis and HCC development in NEMO^LPC-KO^ mice.

### S166A mutation prevents RIPK1-dependent skin inflammation

**Fig. 5:**
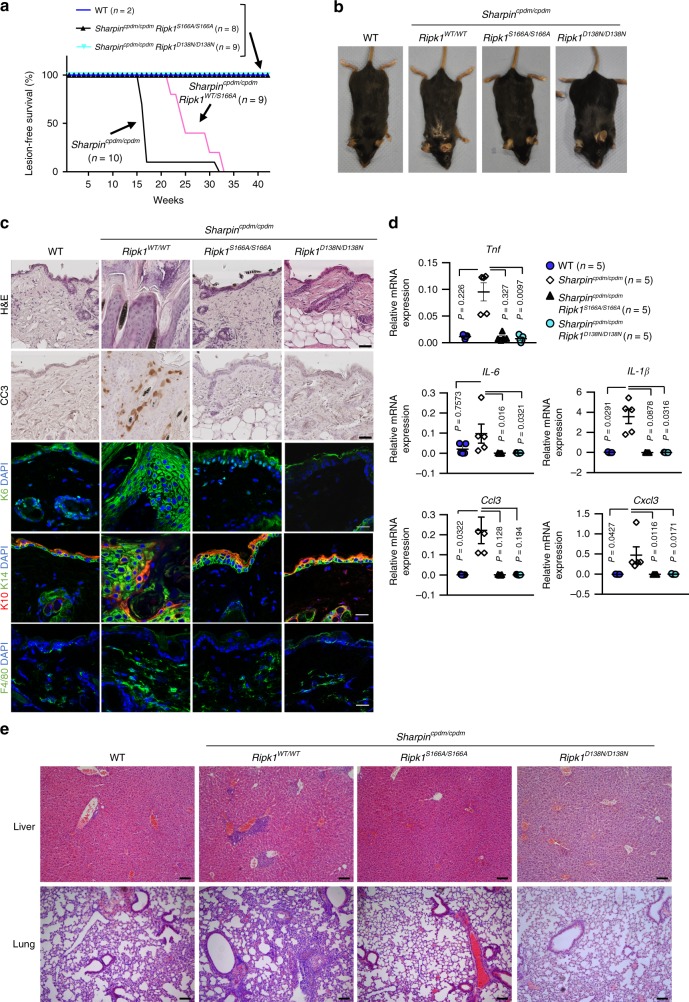
S166 phosphorylation is essential for RIPK1-dependent skin inflammation in *Sharpin*^cpdm/cpdm^ mice. **a** Kaplan-Meier curve of lesion-free survival of mice of the indicated genotypes. **b** Representative macroscopic pictures of mice of the indicated genotypes at the age of 15–17 weeks. **c** Representative skin sections from mice of the indicated genotypes at the age of 15-17 weeks stained with H&E or immunostained with antibodies against cleaved caspase-3 (CC3), keratin 6 (K6), keratin 10 (K10), keratin 14 (K14), F4/80 and DAPI (DNA stain). Scale bars in H&E and CC3 pictures 50 μm, in K6, K10/K14 and F4/80 pictures 20 μm. **d** Graphs depicting relative mRNA expression of the indicated genes in RNA from whole skin tissue of mice of the indicated genotypes at 15–17 weeks of age, measured by qRT-PCR. The dots in the graphs represent individual mice. Mean ± s.e.m. is shown for each group of mice in all graphs. Statistical significance was determined using a Kruskal-Wallis test (one-sided). **e** Representative images of H&E stained tissue sections from liver and lung from mice of the indicated genotypes at 15–17 weeks. Scale bars, 100 μm. Source data for **a**, **d** are provided as a source data file.

Intrigued by the findings that the S166A mutation prevented cell death and inflammation induced by NEMO deficiency in IECs and hepatocytes, we sought to examine the effect of this mutation in other in vivo models of RIPK1 kinase-dependent pathology. SHARPIN-deficient chronic proliferative dermatitis mice (*Sharpin*^*cpdm/cpdm*^) spontaneously develop severe chronic inflammatory skin lesions, accompanied by multi-organ inflammation^50,51^. The skin lesions in *Sharpin*^*cpdm/cpdm*^ mice were previously shown to be driven by TNF-induced FADD-, TRADD- and caspase-8- dependent keratinocyte apoptosis^52,53^, which required RIPK1 kinase activity^13^. We generated *Sharpin*^cpdm/cpdm^ *Ripk1*^*S166A/S166A*^ mice and found that they were fully protected from skin inflammation at least up to the age of 40 weeks, similarly to *Sharpin*^*cpdm/cpdm*^ *Ripk1*^*D138N/D138N*^ mice, in contrast to *Sharpin*^cpdm/cpdm^ mice that developed inflammatory skin lesions between 15–17 weeks of age (Fig. 5a, b). Histological analysis of skin sections from *Sharpin*^*cpdm/cpdm*^ *Ripk1*^*S166A/S166A*^ mice confirmed that the S166A mutation fully prevented keratinocyte apoptosis, epidermal hyperplasia and skin inflammation, similarly to *Sharpin*^cpdm/cpdm^ *Ripk1*^*D138N/D138N*^ mice (Fig. 5c). Furthermore, both the *Ripk1*^*D138N/D138N*^ and the *Ripk1*^*S166A/S166A*^ mutations prevented the upregulation of the mRNA expression of pro-inflammatory cytokines, including *Tnf*, *Il-6*, *IL-1β*, *Ccl3* and *Cxcl3* in the skin of *Sharpin*^*cpdm/cpdm*^ mice (Fig. 5d). In addition, liver and lung inflammation in *Sharpin*^*cpdm/cpdm*^ mice was also inhibited by S166A mutation (Fig. 5e). Thus, RIPK1 autophosphorylation at S166 drives RIPK1 kinase activity-dependent TNFR1-mediated keratinocyte apoptosis and skin inflammation, as well as multi-organ inflammation in *Sharpin*^*cpdm/cpdm*^ mice.

### S166A mutation protects mice from TNF-induced SIRS

**Fig. 6:**
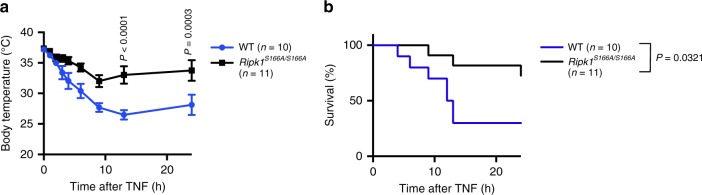
S166 phosphorylation is required for RIPK1-dependent TNF-induced SIRS in mice. **a** Graph shows body temperature of mice of the indicated genotypes that were injected intravenously with 500 μg kg^−1^ body weight. Mean ± s.e.m. is shown for each group of mice. Statistical significance was determined using a two-way ANOVA. **b** Graph shows survival of mice of the indicated genotypes. Statistical significance was determined using a Log-rank (Mantel-Cox) test (two-sided). Source data for **a** and **b** are provided as a source data file.

Injection of a high dose of TNF in mice triggers an acute response often described as systemic inflammatory response syndrome (SIRS), which manifests with hypothermia and increased expression of cytokines and chemokines in the serum and ultimately results in the death of the animals^16^. Previous studies showed that TNF-induced lethality is strongly prevented by RIPK3 deficiency as well as by genetic or pharmacological inhibition of RIPK1 kinase activity^10,16^. Moreover, TNF-induced lethality was partially ameliorated by MLKL deficiency but fully prevented by combined knockout of MLKL and caspase-8 (ref. ^20^), suggesting that acute TNF injection drives systemic pathology by inducing both MLKL-dependent necroptosis and caspase-8-mediated apoptosis. To assess whether RIPK1 autophosphorylation at S166 regulates RIPK1-mediated systemic pathology in response to acute TNF administration, we injected *Ripk1*^*S166A/S166A*^ and WT littermate mice with 500 μg/kg murine TNF intravenously and followed their responses. TNF injection induced the characteristic drop in body temperature in WT mice (Fig. 6a) and resulted in the death of 70% of the animals (Fig. 6b). In contrast, *Ripk1*^*S166A/S166A*^ animals were strongly protected from TNF-induced body temperature reduction (Fig. 6a) and lethality, with only 27% of the mice succumbing (Fig. 6b). Therefore, autophosphorylation of RIPK1 on S166 is critically involved in RIPK1 kinase-dependent systemic toxicity induced by high dose TNF administration.

### S166 phosphorylation enhances the kinase activity of RIPK1

Our results revealed a critical role of RIPK1 autophosphorylation at S166 for the induction of RIPK1 kinase-dependent cell death and inflammation in vivo and in vitro. However, how S166 autophosphorylation affects the capacity of RIPK1 to induce cell death remains elusive. It is generally believed that autophosphorylation induces conformational changes on RIPK1, which allow its interaction with downstream cell death inducers including FADD and RIPK3 and the formation of cell death-inducing signaling complexes. We reasoned that autophosphorylation on S166 could affect RIPK1-mediated assembly of cell death-inducing signaling complexes in two different ways. One possibility is that S166 phosphorylation imposes a conformational change exposing the protein-protein interaction domains of RIPK1, thus facilitating the recruitment of FADD and RIPK3. On the other hand, it is also possible that S166 autophosphorylation enhances the kinase activity of RIPK1, leading to phosphorylation of additional residues such as serine 161 or threonine 169 to induce the conformational changes required for cell death signaling complex assembly.

**Fig. 7:**
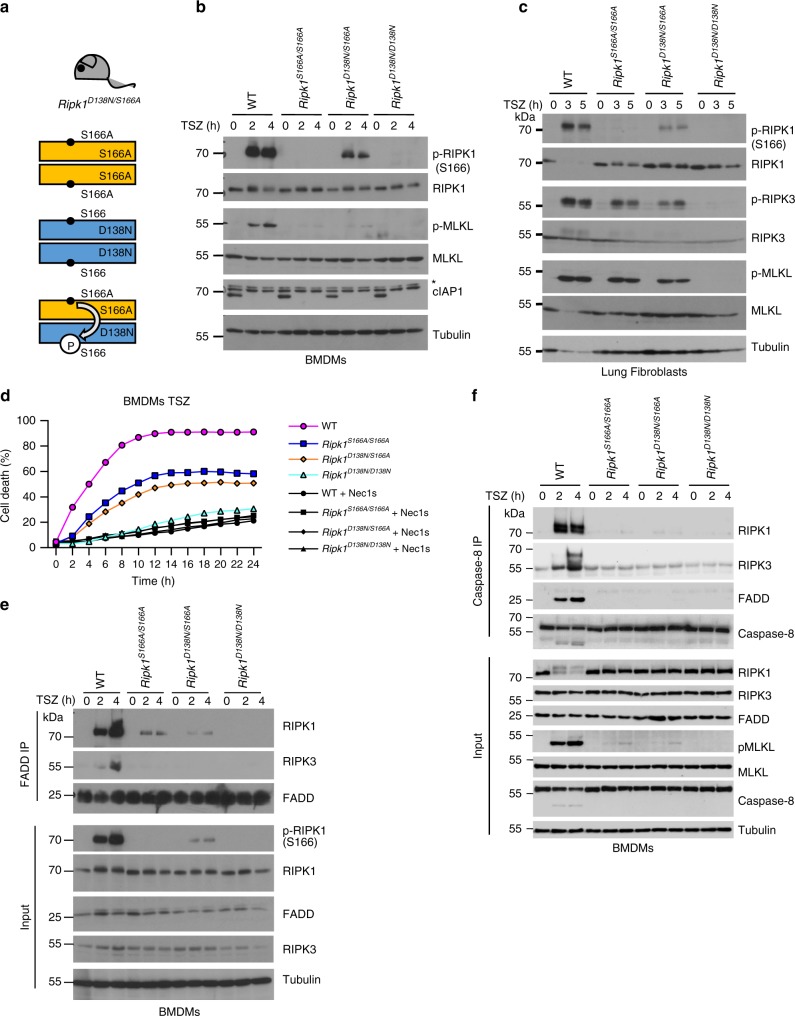
S166 phosphorylation enhances RIPK1 kinase activity. **a** Scheme depicting the possible combinations of RIPK1 dimers in *Ripk1*^*D138N/S166A*^ mice. Residue 166 on RIPK1 is indicated. **b**, BMDMs from mice of the indicated genotypes were treated with a combination of TNF (T; 10 ng ml^−1^), SMAC mimetic (S (Birinapant), 1 μM) and Z-VAD-FMK (Z) for 0, 2 or 4 h and lysates were analyzed by immunoblot with the indicated antibodies. Blots shown are representative of three independent experiments. *Non-specific band. **c** Primary lung fibroblasts from mice of the indicated genotypes were treated with a combination of TNF (T; 20 ng ml^−1^), SMAC mimetic (S (Birinapant), 1 μM) and Z-VAD-FMK (Z) for 0, 3 or 5 h and lysates were analyzed by immunoblot with the indicated antibodies. Blots shown are representative of three independent experiments. **d** BMDMs from mice of the indicated genotypes were treated with combinations of TNF (T; 10 ng ml^−1^), SMAC mimetic (S (Briniapant), 1 μM) and Z-VAD-FMK (Z) in the presence or absence of Nec-1s. Cell death was measured using IncuCyte. Graph shows mean of technical triplicates. Data are representative of three independent experments. For purpose of comparability the data of the genotypes WT, *Ripk1*^*S166A/S166A*^ and *Ripk1*^*D138N/D138N*^ shown here are identical to those in Fig. 1b. **e** BMDMs were treated with a combination of TNF (T; 10 ng ml^−1^), SMAC mimetic (S (Birinapant), 1 μM) and Z-VAD-FMK (Z) for 0, 2 or 4 h. FADD immunoprecipitates and total cell lysates (input) were analyzed by immunoblot using the indicated antibodies. **f** BMDMs were treated with a combination of TNF (T; 10 ng ml^−1^), SMAC mimetic (S (Birinapant), 1 μM) and Z-VAD-FMK (Z, 50 μM) for 0, 2 or 4 h. Caspase-8 immunoprecipitates and input were analyzed by immunoblot using the indicated antibodies. Source data for **d** are provided as a source data file.

In order to distinguish between these two possible functions of S166 autophosphorylation, we designed an experiment allowing to assess whether the S166A mutation affects the kinase activity of RIPK1 and also if S166 phosphorylation is the critical step inducing the conformational changes of RIPK1 required for the induction of cell death. In designing this experiment, we wanted to use the most physiological system possible, as overexpression and reconstitution experiments in cell lines are known to often result in artifacts. To this end, we generated *Ripk1*^*D138N/S166A*^ mice expressing the kinase inactive RIPK1D138N protein from one allele and the RIPK1S166A protein from the other allele, ensuring that both proteins are expressed at physiological levels. In these *Ripk1*^*D138N/S166A*^ cells, three different dimers of RIPK1 can form: RIPK1D138N and RIPK1S166A homodimers, as well as RIPK1D138N/RIPK1S166A heterodimers (Fig. 7a). The formation of RIPK1D138N/RIPK1S166A heterodimers allows to assess whether RIPK1S166A exhibits altered kinase activity by examining the phosphorylation of the RIPK1D138N protein on S166 using specific antibodies recognizing phosphorylated S166. We therefore stimulated BMDMs or LFs from WT, *Ripk1*^*S166A/S166A*^, *Ripk1*^*D138N/S166A*^ and *Ripk1*^*D138N/D138N*^ animals with TNF in the presence of Z-VAD-FMK and birinapant (TSZ) and assessed S166 phosphorylation by immunoblotting two and four hours after stimulation. As shown in Fig. 7b, c, strong phosphorylation of S166 was detected in TSZ-stimulated WT cells, which was fully abolished in cells from *Ripk1*^*D138N/D138N*^ and *Ripk1*^*S166A/S166A*^ mice. Importantly, S166 phosphorylation was detected in *Ripk1*^*D138N/S166A*^ cells, showing that RIPK1S166A exhibits kinase activity and can phosphorylate RIPK1D138N (Fig. 7b, c). However, phosphorylation of S166 was strongly reduced in cells from *Ripk1*^*D138N/S166A*^ mice in comparison to WT cells, suggesting that RIPK1S166A exhibits reduced kinase activity (Fig. 7b, c). Of note, cIAP1 was non-detectable in response to TSZ treatment, indicating that birinapant led to full degradation of the protein (Fig. 7b). The results from this experiment, which represents an in vivo kinase assay, revealed that RIPK1S166A exhibited kinase activity and was capable to phosphorylate RIPK1D138N, albeit with reduced efficiency (Fig. 7b, c).

We then wanted to address whether S166 phosphorylation is a critical event in imposing the conformational change required for the RIPK1-dependent assembly of the cell death complex and the induction of cell death. If that were the case, we would expect that phosphorylation of RIPK1D138N at S166 would render this kinase-inactive RIPK1 molecule capable of inducing cell death. We therefore assessed the induction of necroptosis in BMDMs from *Ripk1*^*D138N/S166A*^ mice in comparison to WT, *Ripk1*^*D138N/D138N*^ and *Ripk1*^*S166A/S166A*^ cells. As shown in Fig. 7d, TSZ stimulation induced similar levels of cell death in *Ripk1*^*D138N/S166A*^ compared to *Ripk1*^*S166A/S166A*^ BMDMs both in terms of kinetics and the amount of dying cells. Importantly, *Ripk1*^*D138N/S166A*^ BMDMs did not show increased cell death compared to *Ripk1*^*S166A/S166A*^ cells, despite the fact that S166 phosphorylation was detected under these conditions. Consistent with the cell death assay results, phosphorylation of RIPK3 and MLKL were reduced to a similar extent in TSZ-stimulated LFs from *Ripk1*^*D138N/S166A*^ and *Ripk1*^*S166A/S166A*^ mice (Fig. 7c). Furthermore, immunoprecipitation with anti-FADD or anti-caspase-8 antibodies and subsequent immunoblotting revealed strongly impaired recruitment of RIPK1 and RIPK3 to the necrosome in both *Ripk1*^*D138N/S166A*^ and *Ripk1*^*S166A/S166A*^ BMDMs in response to TSZ stimulation (Fig. 7e, f). Taken together, these results showed that phosphorylation of RIPK1D138N at S166 in *Ripk1*^*D138N/S166A*^ cells did not increase the efficiency of necrosome formation and the induction of necroptosis compared to *Ripk1*^*S166A/S166A*^ cells. Our interpretation of these findings is that autophosphorylation at S166 is not sufficient to trigger the conformational changes required for cell death induction by RIPK1.

### S166A inhibits RIPK1 autophosphorylation at other sites

**Fig. 8:**
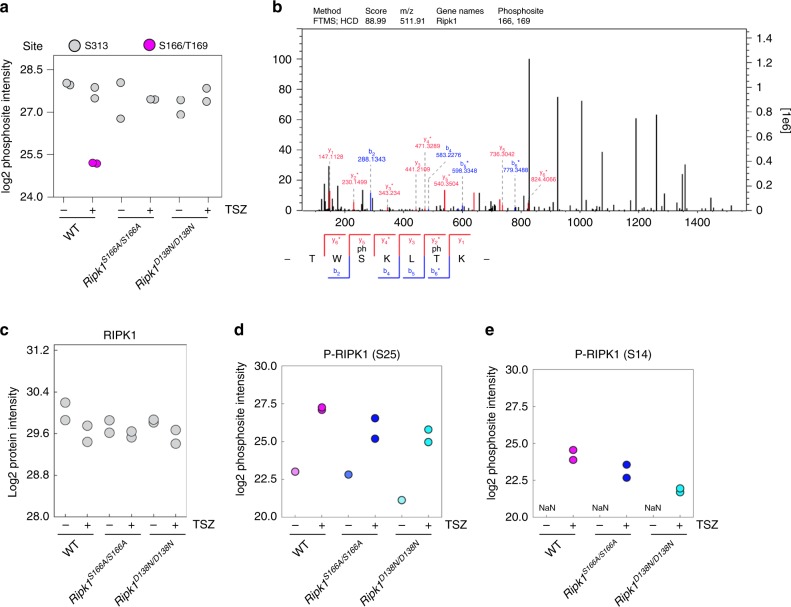
S166 phosphorylation promotes RIPK1 autophosphorylation at additional sites. **a** LC–MS/MS measured log2 intensities of the p-S166 p-T169- and p-S313 sites after indicated treatment with or without a combination of TNF (10 ng ml^−1^), SMAC mimetic ((Birinapant), 1 μM) and Z-VAD-FMK (TSZ) for 2 h. **b** Selected LC–MS/MS scan of the p-S166/p-T169 RIPK1 peptide with annotated b-and y-ions. Fragment ions marked with asterisk (*) result from loss of the phospho-group. **c** LC–MS/MS measured log2 intensities of RIPK1 peptides after indicated treatment with or without a combination of TNF (10 ng ml^−1^), SMAC mimetic ((Birinapant), 1 μM) and Z-VAD-FMK (TSZ) for 2 h. **d** LC–MS/MS measured log2 intensities of the RIPK1 p-S25 site after indicated treatment with or without a combination of TNF (10 ng ml^−1^), SMAC mimetic ((Birinapant), 1μM) and Z-VAD-FMK (TSZ) for 2 h. **e** LC–MS/MS measured log2 intensities of the RIPK1 p-S14 site after indicated treatment with or without a combination of TNF (10 ng ml^−1^), SMAC mimetic ((Birinapant), 1μM) and Z-VAD-FMK (TSZ) for 2 h. **a**, **c**–**e**, Values from two independent experiments are shown.

Besides S166, mouse RIPK1 has been shown to autophosphorylate at S14, S15 and S161, as well as T169 (refs. ^32,37^) indicating that multiple autophosphorylation events might be required to unfold the full cytotoxic potential of RIPK1. To explore the impact of RIPK1S166A mutation on RIPK1 autophosphorylation at other sites, we analyzed RIPK1 phosphorylation in TSZ-treated BMDMs by mass spectrometry (MS). After phospho-peptide enrichment with titanium dioxide (TiO_2_) beads, we found in total 13,547 phosphorylation sites, including 5 sites on RIPK1. Interestingly, we measured a peptide containing double phosphorylation at S166 and T169 (p-S166/p-T169) after two hours of TSZ treatment in WT cells (Fig. 8a, b). This peptide was not found in *Ripk1*^*D138N/D138N*^ cells, therefore also T169 likely represents an autophosphorylation site. Importantly, p-T169 was not detected in *Ripk1*^*S166A/S166A*^ cells, suggesting that S166 phosphorylation might be required for subsequent phosphorylation of T169. We detected relatively equal levels of total RIPK1 protein as well as RIPK1 phosphorylation at S313 under all conditions (Fig. 8a, c), indicating that phospho-peptide enrichment was consistently efficient between the different samples. Furthermore, we detected phosphorylation of S25 of RIPK1 in all genotypes (Fig. 8d), consistent with previous studies showing that S25 is phosphorylated by IKKs^32^. In addition, we found RIPK1 to be phosphorylated at S14 (Fig. 8e), which was previously suggested to be an autophosphorylation site^37^. S14 phosphorylation was detected also in *Ripk1*^*S166A/S166A*^ cells, although at reduced levels compared to wild-type cells (Fig. 8e). Surprisingly, low levels of S14 phosphorylation were measured in *Ripk1*^*D138N/D138N*^ cells that lack RIPK1 kinase activity (Fig. 8e), suggesting that S14 is not exclusively a RIPK1 autophosphorylation site. Earlier studies reported RIPK1 autophosphorylation at S161 in response to necroptotic stimuli^37,38^. However within two independent experiments we did not detect phosphorylation of S161 in TSZ-treated BMDMs, although in these replicates as well as in a broader context of MS experiments targeted to RIPK1, we reproducibly measured the non-phosphorylated peptide. Collectively, these results suggest that autophosphorylation at S166 promotes the autophosphorylation of additional sites on RIPK1.

## Discussion

RIPK1 kinase activity has emerged as a key regulator of cell death and inflammation that contributes to the pathogenesis of inflammatory diseases, but the mechanisms by which RIPK1 kinase activation triggers downstream signaling remain poorly understood. Previous experiments using primarily overexpression and reconstitution approaches in cell lines suggested that phosphorylation of S161 is the critical event triggering RIPK1 kinase activity-dependent cell death^38^; however, the role of S166 phosphorylation has not been assessed. Here we identify autophosphorylation at S166 as a critical event regulating RIPK1-mediated apoptosis and necroptosis, as well as the development of inflammatory pathologies in vivo. Our experiments in *Ripk1*^*S166A/S166A*^ knock-in mice revealed that abolishing phosphorylation at S166 prevented the development of RIPK1 kinase-dependent skin, intestinal, liver and systemic inflammatory pathologies in relevant mouse models. Interestingly, the S166A mutation did not only inhibit TNF-mediated inflammation in the *Sharpin*^cpdm/cpdm^, NEMO^IEC-KO^ and TNF-induced SIRS mouse models, but also fully prevented liver pathology in NEMO^LPC-KO^ mice, which develops independently of TNFR1, Fas and TRAILR^49^. Therefore, S166 phosphorylation regulates both TNF-dependent and -independent RIPK1 kinase activity-driven inflammatory pathologies.

Our in vitro experiments showed that cells expressing RIPK1S166A were partially, but not completely protected from TNF-induced apoptosis and necroptosis as well as TLR3- and TLR4-induced necroptosis. In contrast, our in vivo studies in three different genetic mouse models of RIPK1-mediated pathologies showed that the S166A mutation was as effective as the D138N mutation in preventing inflammation. Therefore, under physiological conditions that are best modeled in vivo, S166 phosphorylation is essential for activating the cytotoxic potential of RIPK1. These findings also question the physiological significance of in vitro stimulation experiments exposing cells to high concentrations of TNF and other cytotoxic stimuli over several hours, which are unlikely to occur in the context of a whole organism, and argue that such results should be treated with caution with regards to their in vivo relevance.

Our experiments provided important mechanistic insights into the molecular function of S166 phosphorylation for the activation of RIPK1 kinase-dependent signaling. Using an innovative approach for the assessment of RIPK1 kinase activity under physiological protein concentrations within primary cells from *Ripk1*^*D138N/S166A*^ mice, we found that mutation of S166 inhibited, but could not abolish, RIPK1 kinase activity. This finding suggests that S166 autophosphorylation acts as an amplification step to unleash the full activation of the kinase. Moreover, our results showing that RIPK1S166A can phosphorylate RIPK1D138N on S166 under endogenous expression levels provided experimental evidence that RIPK1 autophosphorylation occurs in trans within a dimer, as suggested previously based on artificial dimerization systems^22^. Furthermore, our experiments in primary cells from *Ripk1*^*D138N/S166A*^ mice showed that the presence of RIPK1D138N molecules phosphorylated on S166 did not cause increased cell death, arguing that S166 phosphorylation is not sufficient by itself to impose the conformational changes required for RIPK1 to engage and activate the apoptosis- and necroptosis-inducing cell death machineries. Together, these results argue that S166 phosphorylation licenses the autophosphorylation of additional sites on RIPK1, which are then required either individually or in combination to facilitate the interaction of RIPK1 with downstream effectors and the activation of cell death.

In addition to S166, serine residues at position 14, 15 and 161 as well as threonine at position 169 have been previously identified as autophosphorylation sites of mouse RIPK1 (refs. ^13,32,37,38^). Although autophosphorylation at S161 was identified and proposed to have a critical role in RIPK1-mediated cell death in studies performed in cell lines overexpressing RIPK1 constructs^37,38^, we did not detect phosphorylation of endogenous RIPK1 at S161 in our experiments. Whereas our inability to detect S161 phosphorylation may be related to the specific experimental setup, in our assays we clearly measured phosphorylation of S14, S166 and S169 of endogenous RIPK1 in primary BMDMs arguing against insufficient activation of RIPK1. Moreover, we could reproducibly measure the un-phosphorylated peptide containing S161, arguing against problems with peptide detection. Together, these results question the possible role of S161 autophosphorylation in regulating RIPK1 kinase activity-dependent functions. Further studies will be required to experimentally validate the possible role of S161 and other RIPK1 autophosphorylation sites in regulating RIPK1-dependent functions. Particularly taking into account our findings that abolishing S166 phosphorylation could fully prevent RIPK1 kinase-dependent cell death and inflammation in vivo, but only partially prevented RIPK1 kinase-mediated cell death in cell culture assays, it will be crucial to assess the role of different autophosphorylation sites in a physiological setting by generating mouse models expressing endogenous RIPK1 with specific mutations. It is also possible that different sites may show functional redundancy, therefore generating mouse models with mutations of different sites in combination would be necessary in order to unravel the mechanisms by which autophosphorylation regulates the activation of RIPK1 kinase-dependent cell death.

Taken together, our results identify S166 autophosphorylation as an essential event for the activation of RIPK1-dependent cell death and the pathogenesis of inflammatory pathologies in vivo in relevant mouse models. These findings support the important role of S166 phosphorylation as a biomarker for RIPK1-mediated pathologies, which will be crucial in order to better stratify human patients to identify those that are more likely to benefit from treatment with RIPK1 inhibitors that reached phase II clinical trials^24,25^.

## Methods

### Mice

*Ripk1*^*D138N/D138N*^^43^, *Nemo*^*FL*^^54^, *Villin-Cre*^55^, *Alfp-Cre*^56^ and *Sharpin*^*cpdm/cpdm*^^51^ were described previously. Mice were maintained at the SPF animal facilities of the Institute for Genetics and the CECAD Research Center of the University of Cologne, under a 12 h light cycle, at a temperature of 22 ± 2 °C, 55 ± 5% relative humidity and given a regular chow diet (Harlan, diet number 2918 or Prolab Isopro RMH3000 5P76) *ad libitum*. All animal procedures were conducted in accordance with European, national and institutional guidelines and protocols were approved by the responsible local authorities (Landesamt für Natur, Umwelt und Verbraucherschutz Nordrhein-Westfalen, Germany; ethical committee of Ghent University, Belgium). Animals requiring medical attention were provided with appropriate care and were sacrificed when they developed macroscopically visible skin lesions to minimize suffering. Mice of the indicated genotype were assigned at random to groups. Mouse studies were performed in a blinded fashion.

### CRISPR/Cas9-mediated generation of *Ripk1*^*S166A/S166A*^ mice

For the generation of *Ripk1*^*S166A/S166A*^ mice, Cas9 mRNA (TriLink) together with the 120 bp ssDNA repair oligo (IDT) and the short guide RNA (sgRNA) were microinjected into the pronucleus of fertilized oocytes obtained from C57BL/6 mice. A sgRNA targeting a site adjacent to position 166 of the murine *Ripk1* gene was used (5’ GAC ATG GAG CAA ACT GAC TA3’). On the next day, the injected embryos were transferred to foster mothers and allowed to develop to term. Mutations in the genome of progeny were determined by analysis of genomic DNA using the T7 endonuclease I assay (NEB) and sequencing. Sequenced DNA fragments were aligned using Benchling [Biology Software] (2020, https://benchling.com). The sequence of the ssDNA oligo used as repair template for the RIPK1S166A knock-in was 5ʹ CTC TTC TTT TCC AGA TAG CCG ATC TTG GTG TGG CTT CCT TTA AGA CAT GGG CCA AA C TGA CTA AAG AGA AAG ACA ACA AGC AGA AAG AAG TGAG CAG CAC CAC TAA GAA GAA CAA TGG TG 3ʹ. Primers to amplify DNA fragments for Surveyor assay and sequencing were: (5ʹ GCA GCC ACT GGA AAA TTG AT 3ʹ) and (5ʹ TGT CTT ACT CTC ATA GGG CTC C 3ʹ).

### Histological analysis of tissue sections

Skin, colon and liver tissue samples from mice were fixed overnight in 4% paraformaldehyde, embedded in paraffin, and cut in 3–5 µm sections. For Immunohistochemistry staining, paraffin sections were rehydrated and heat-induced antigen retrieval was performed in Tris-citrate buffer. Sections were incubated with primary antibodies against cleaved caspase-3 (9661, Cell Signaling Technology), Ki-67 (DAKO, M724901), α-SMA (Sigma-Aldrich, A2547), F4/80 (clone A3-1, AbD Serotec), Keratin 14 (MS-115, Neomarkers), Keratin 6 (PRB-169P, Covance) and Keratin 10 (PRB-159P, Covance). Biotinylated secondary antibodies were purchased from Perkin Elmer, Dako and Invitrogen. Nuclei were stained by DAPI (VectorLabs). Stainings were visualized with ABC Kit Vectastain Elite (Vector Laboratories) and DAB substrate (Dako and Vector Laboratories) or Alexa-488 (A1101, Molecular Probes) and Alexa-549 (A11012, Molecular Probes)-fluorescence-conjugated secondary antibodies. F4/80 staining was done on cryo sections.

### Histopathological scoring

Histopathological evaluation of colon tissue sections was performed on 3 µm H&E stained intestinal tissue sections in a blinded fashion as described previously^57^. In brief, inflammation was assessed by the presence and the localization of inflammatory immune cell infiltrates (0: absent, 1: mucosal, 2: submucosal, 3: transmural extending into muscularis and serosa, and 4: diffuse). For the evaluation of tissue damage four scores were ascribed to crypt hyperplasia, epithelial injury and death of epithelial cells (0: absent, 1: mild, 2: moderate and 3: severe). The sum of these inflammation or tissue damage scores were then multiplied by a factor according to the fraction of the tissue being affected: 1: < 25%; 2: 25–50%; 3: 50–70% and 4: >75%. The total histological score represents the sum of inflammation and damage marks. Percentages of cleaved caspase-3 positive crypts were determined by counting the number of crypts containing cleaved caspase-3 positive cells in a representative area of the section and normalizing to the total number of crypts in that area.

### Primary cell isolation and culture

Lung fibroblasts were prepared from whole lung tissue and dermal fibroblasts were prepared from tail and ear tissue using Collagenase Type 2 (Worthington, 44N15307B). Fibroblasts were maintained in Dulbecco’s Modified Eagle Medium (Gibco) supplemented with Penicillin-Streptomycin, l-Glutamine, Sodium Pyruvate, HEPES Buffer Solution (1% each, all Gibco) and 10% fetal serum (PAN Biotech). BMDMs were isolated from mice according to standard procedures and maintained in similar medium as fibroblasts, but with 10% fetal bovine serum superior (Biochrom) and 20 ng ml^−1^ M-CSF (Immunotools, 12343118). BMDMs were differentiated for 6 days before plating for experiments.

### Immunoblotting and immunoprecipitation

Antibodies against the following proteins were used for immunoblot analysis: p-IκBα (2859, Cell Signaling Technology), IκBα (sc-371, Santa Cruz Biotechnology), p-p65 (3033, Cell Signaling Technology), p65 (sc-372, Santa Cruz Biotechnology), p-SAPK/JNK (T183/T185) (4668, Cell Signaling Technology), SAPK/JNK (9252, Cell Signaling Technology), p-p38 (9211, Cell Signaling Technology), p38 (9212, Cell Signaling Technology), p-p44/42 MAPK/ERK (9191, Cell Signaling Technology), p44/42 MAPK (9102, Cell Signaling Technology), RIPK1 (610459, BD or 3493, Cell Signaling Technology, or custom-made rabbit anti-RIPK1 serum), p-RIPK1(S166) (31122, Cell Signaling Technology), p-RIPK3 (57220, Cell Signaling Technology), RIPK3 (ADI-905-242-100, Enzo Life Sciences or clone1G6.14, Genentech^58^ or PA5-19956, Thermo Fisher), p-MLKL (S345; D6E3G, Cell Signaling Technology or ab196436, Abcam), MLKL (MABC604, Millipore), caspase-8 (ALX-804-447, Alexis), FADD (05-486, upstate or sc-6036, Santa Cruz Biotechnology or ADI-AAM-212-E, Enzo Life Scienes), TRADD (AHP2533, Bio-Rad Laboratories), GAPDH (NB300-221, Novus Biologicals), Tubulin (T6074, Sigma-Aldrich or ab21058, Abcam), mouse IgG HRP-linked antibody (NA931, GE Healthcare), rabbit IgG HRP-linked antibody (NA934V, GE Healthcare) and rat conjugated to HRP (112-035-003, Jackson Immuno Research). Signals were detected using SuperSignal West Pico Chemiluminescent substrate (34080, Thermo Fisher Scientific), Amersham ECL Western Blotting Detection Reagent (GE Healthcare) or SuperSignal™ West Pico PLUS Chemiluminescent Substrate (34580, Thermo Fisher Scientific) and SuperSignal™ West Femto (34095, Thermo Fisher Scientific). The membranes were reprobed after incubation in Restore Western Blot stripping buffer (21059, Thermo Fisher Scientific). To check RIPK1-interacting proteins, RIPK1 was immunoprecipitated using custom-made anti-RIPK1 serum which was BS3-crosslinked to Dynabeads™ Protein G (10009D, Invitrogen). In brief, cells were placed on ice after treatment and cell lysates were prepared in immunoprecipitation (IP) buffer (20 mM HEPES-KOH (pH 7.6), 150 mM NaCl, 2 mM EDTA, 1% Triton X-100, 10% Glycerol, cOmplete Tablets Mini (Roche, 04693124001) and PhosSTOP (Roche, 04906837001). Crosslinked beads and serum were added to lysates and left overnight with rotation at 4 °C. The next day, beads were washed twice in IP buffer and immunprecipitates were eluted by boiling in Laemmli buffer. For FADD-IPs and caspase-8 IPs, the protocol was similar, however in this case Protein G beads (Invitrogen for FADD IPs and 10003D, Life Technologies for caspase-8 IPs) were pre-incubated with anti-FADD antibody (sc-6036, Santa Cruz Biotechnology) or anti-caspase-8 custom-made serum without cross-linking and subsequently added to cell lysates for incubation overnight. For TNFR1 complex I IPs, 8×10^6^ BMDMs were seeded on a 60 cm^2^ tissue culture plate. The next day, cells were treated with 1 µg ml^−1^ FLAG-hTNF. The cells were washed two times in ice-cold PBS, followed by lysis in NP-40 lysis buffer (10% glycerol, 1% NP-40, 150 mM NaCl and 10 mM Tris-HCl pH 8 supplemented with phosphatase and protease inhibitor cocktail tablets (Roche Diagnostics)). The cell lysates were cleared by centrifugation for 15 min at 4 °C and the supernatants were then incubated overnight with FLAG M2 affinity gel (Sigma-Aldrich). The next day, the beads were washed three times in NP-40 lysis buffer and FLAG-eluted using 150 ng µl^−1^ 3X FLAG peptide for 30 min at RT. The FLAG eluate was diluted in 5x Laemmli buffer and analyzed by immunoblotting.

### Cell death assays

BMDMs were seeded at 25.000 cells, dermal and lung fibroblasts were seeded at 10.000 cells per well in a 96 well plate. The following day, cells were pre-treated with combinations of Z-VAD-FMK (Enzo, ALX-260-020-M005, 20 μM if not specified otherwise), Nec1s (BioVision, 2263, 20 μM), Birinapant (BioVision, 2597) in the presence of the dead cell stain Yoyo^TM^-1 Iodide (Yoyo-1; Invitrogen, Y3601; 0,5 μM for fibroblasts and 0,25 μM for BMDMs) for 30 min, before addition of recombinant mouse TNF (VIB Protein Service Facility, Ghent), LPS (Enzo, ALX-581-010-L002, 100 ng ml^−1^) or Poly (I:C) (EDM Millipore, 42424-50-0, 50 μg ml^−1^ for analysis of NF-κB and MAPK activation, 0.5 μg ml^−1^ for cell death assays). Dead cells were imaged in real-time for 24 h in intervals of 2 h via fluorescence signals using an IncuCyte® S3 Live-Cell Analysis System (Essen Bioscience). Percentages of cell death were calculated by treating two wells per genotype in each experiment with the cell-permeable fluorescent stain Draq5 (Cell Signaling Technology, #4084L, 500 μM) and imaging at a 0 h timepoint, which allows quantification of the total number of cells present per field. Resulting images were analyzed using the software package of the IncuCyte which allows quantification of the number of Yoyo-1 or Draq5-positive cells. Dead cell counts acquired via Yoyo1-1 staining were divided by the total cell number of Draq5-positive cells to yield the percentage of cell death at each timepoint. Curves show the mean of two technical replicates.

### Quantitative RT-PCR

Total RNA was extracted using Trizol Reagent (Life Technologies) and RNeasy Columns (Qiagen) followed by cDNA preparation using Superscript III cDNA-synthesis Kit (Life technologies). qRT-PCR was performed using TaqMan probes in duplicates for each sample (Life technologies) with *Hprt* or *Tbp* serving as reference gene. Relative expression of gene transcripts was analyzed via the 2-ΔCt method and are presented in dot plot graphs as mRNA expression values relative to the reference gene.

### RNA isolation and microarray analysis

Colon tissues were disrupted using a Precellys 24 (Bertin technologies) and total RNA from colon tissue was extracted using a Nucleospin® RNA kit (Macherey-Nagel) according to manufacturer’s instruction. 100 ng of total RNA per sample were used as input for the Clariom-S-mouse microarray (Thermo Fisher Scientific) analysis. Target preparation was performed using the Gene Chip^TM^ WT PLUS Reagent Kit (Thermo Fisher Scientific) according to manufacturer’s instructions. Hybridization was performed in a Gene Chip Hybridization Oven 645 for 16 h at 45 °C. Gene chips were scanned using the Gene Chip Scanner 7 G. The Clariom-S-mouse array was in a 169 format, for which the Fluidics Protocol FS450-0007 was used. Array-Quality Control was done using the Affymetrix Expression Console or Transcriptome Analysis Console (TAC) Software. Z score transformation and heat cluster data analysis was performed using InstantClue^59^.

### TNF-induced SIRS model

For the TNF shock model, 500 µg kg^−1^ mTNF (diluted in endotoxin-free PBS pH 6.8) was intravenously injected in mice. Mortality and body temperature were monitored until 36 h after mTNF injection. Rectal body temperature was recorded with an industrial electric thermometer (Comark Electronics, Norwich, UK; model 2001). Mice were sacrificed when body temperature reached 25 °C.

### LC–MS/MS phosphorylation analysis

Cell lysates were precipitated with acetone and resuspended in 8 M Urea. After reduction with DTT and carbamidomethylation with IAA, proteins were digested overnight at RT, using the proteases LysC (Wako) and trypsin (Promega). Samples were desalted using SepPak C18 cartridges (Waters) and phosphorylated peptides were enriched with the High-Select TiO2 Phosphopeptide Enrichment Kit (Thermo Fisher Scientific). Proteomic analysis was performed using an Easy nLC 1000 UHPLC coupled to a QExactive Plus mass spectrometer (Thermo Fisher Scientific). Peptides were resuspended in Solvent A (0.1% FA), picked up with an autosampler and loaded onto in-house made 50 cm fused silica columns (internal diameter (I.D.) 75 μm, C18 1.7 μm, Dr. Maisch beads) at a flow rate of 0.75 µl min^−1^. A 90 min segmented gradient of 8-48% Solvent B (80% ACN in 0.1% FA) over 61 min, 48-70% Solvent B over 6 min, and 70-95% Solvent B over 5 min at a flow rate of 250 nl min^−1^ was used to elute peptides. Eluted peptides were sprayed into the heated transfer capillary of the mass spectrometer using a nano-electrospray ion source (Thermo Fisher Scientific). The mass spectrometer was operated in a data-dependent mode, where the Orbitrap acquired full MS scans (300-1750 *m*/*z*) at a resolution (R) of 70,000 with an automated gain control (AGC) target of 3×10^6^ ions collected within 20 ms. The dynamic exclusion time was set to 20 s. From the full MS scan, the 10 most intense peaks (*z* ≥ 2) were fragmented in the high-energy collision-induced dissociation (HCD) cell. The HCD normalized collision energy was set to 25%. MS/MS scans with an ion target of 1×10^6^ ions were acquired with *R* = 17,500, with a maximal injection time of 80 ms and an isolation width of 2.0 *m*/*z*. The raw files were processed using MaxQuant software (Version 1.5.3.8.) and its implemented Andromeda search engine^60^. Minimum score for modified peptides was set to 0, remaining parameters were set to default values and Phospho (STY) was added as variable modification. Phosphosite intensities were logarithmized and gene ontology annotations assigned using Perseus software^61^.

### Statistics and reproducibility

When data fulfilled the criteria for Gaussian distribution, one-way Anova was performed; otherwise the nonparametric one-sided Kruskal-Wallis test was chosen. Statistical analysis was performed with Prism, GraphPad. Histological analysis was performed independently on tissue samples from at least three animals per genotype. Specifically, in Fig. 4b: WT *n* = 3, NEMO^LPC-KO^ *n* = 3, NEMO^LPC-KO^ *Ripk1*^*S166A/S166A*^ *n* = 5, *Ripk1*^*S166A/S166A*^ *n* = 3, in Fig. 4d: WT *n* = 3, NEMO^LPC-KO^ *n* = 3, NEMO^LPC-KO^ *Ripk1*^*S166A/S166A*^ *n* = 5, *Ripk1*^*S166A/S166A*^ *n* = 3 in Fig. 5c *n* = 5 (H&E) or *n* = 3 (CC3, K10/14, K6, F4/80) per genotype and in Fig. 5e *n* = 3 samples per genotype were examined.

### Reporting Summary

Further information on research design is available in the Nature Research Reporting Summary linked to this article.

## Supplementary information

Supplementary Information

Reporting Summary

## Data Availability

The microarray data discussed in this publication have been deposited in NCBI’s Gene Expression Omnibus^62^ and are accessible through GEO Series accession number GSE131855. The mass spectrometry proteomics data have been deposited to the ProteomeXchange Consortium via the PRIDE^63^ partner repository with the dataset identifier PXD014097. The source data underlying Figs. 1b–i, 3b–d, 4a, e, f, 5a, d, 6a, b, and 7d and Supplementary Fig. 1d are provided as a Source Data file. Uncropped images of immunoblots presented in the figures are included in “Supplementary Fig. 2”.

## Source Data

Source Data

## References

[1] Degterev A, Ofengeim D, Yuan J (2019). Targeting RIPK1 for the treatment of human diseases. Proc. Natl. Acad. Sci. USA.

[2] Kondylis V, Pasparakis M (2019). RIP Kinases in Liver Cell Death, Inflammation and Cancer. Trends Mol. Med..

[3] Dannappel M (2014). RIPK1 maintains epithelial homeostasis by inhibiting apoptosis and necroptosis. Nature.

[4] Dillon CP (2014). RIPK1 blocks early postnatal lethality mediated by caspase-8 and RIPK3. Cell.

[5] Kaiser WJ (2014). RIP1 suppresses innate immune necrotic as well as apoptotic cell death during mammalian parturition. Proc. Natl Acad. Sci. USA.

[6] Kelliher MA (1998). The death domain kinase RIP mediates the TNF-induced NF-kappaB signal. Immunity.

[7] Rickard JA (2014). RIPK1 regulates RIPK3-MLKL-driven systemic inflammation and emergency hematopoiesis. Cell.

[8] Takahashi N (2014). RIPK1 ensures intestinal homeostasis by protecting the epithelium against apoptosis. Nature.

[9] Lin J (2016). RIPK1 counteracts ZBP1-mediated necroptosis to inhibit inflammation. Nature.

[10] Newton K (2016). RIPK1 inhibits ZBP1-driven necroptosis during development. Nature.

[11] Pasparakis M, Vandenabeele P (2015). Necroptosis and its role in inflammation. Nature.

[12] Newton K. Multitasking kinase RIPK1 regulates cell death and inflammation. *Cold Spring Harb. Perspect. Biol.* **12**, (2019). 10.1101/cshperspect.a036368 PMC7050590 31427374

[13] Berger SB (2014). Cutting edge: RIP1 kinase activity is dispensable for normal development but is a key regulator of inflammation in SHARPIN-deficient mice. J. Immunol..

[14] Kondylis V (2015). NEMO prevents steatohepatitis and hepatocellular cacinoma by inhibiting RIPK1 kinase activity-mediated hepatocyte apoptosis. Cancer Cell.

[15] Vlantis K (2016). NEMO prevents RIP kinase 1-mediated epithelial cell death and chronic intestinal inflammation by NF-kappaB-dependent and -independent functions. Immunity.

[16] Duprez L (2011). RIP kinase-dependent necrosis drives lethal systemic inflammatory response syndrome. Immunity.

[17] Polykratis A (2019). A20 prevents inflammasome-dependent arthritis by inhibiting macrophage necroptosis through its ZnF7 ubiquitin-binding domain. Nat. Cell Biol..

[18] Chen Y (2018). Necrostatin-1 improves long-term functional recovery through protecting oligodendrocyte precursor cells after transient focal cerebral ischemia in mice. Neuroscience.

[19] Naito MG (2020). Sequential activation of necroptosis and apoptosis cooperates to mediate vascular and neural pathology in stroke. Proc. Natl Acad. Sci. USA.

[20] Newton K (2016). RIPK3 deficiency or catalytically inactive RIPK1 provides greater benefit than MLKL deficiency in mouse models of inflammation and tissue injury. Cell Death Differ..

[21] Ofengeim D (2015). Activation of necroptosis in multiple sclerosis. Cell Rep..

[22] Xu D (2018). TBK1 suppresses RIPK1-driven apoptosis and inflammation during development and in aging. Cell.

[23] Ofengeim D (2017). RIPK1 mediates a disease-associated microglial response in Alzheimer’s disease. Proc. Natl Acad. Sci. USA.

[24] Weisel K., et al. Randomized clinical study of safety, pharmacokinetics, and pharmacodynamics of RIPK1 inhibitor GSK2982772 in healthy volunteers. *Pharmacol. Res. Perspect.* **5**, (2017). 10.1002/prp2.365 PMC5723699 29226626

[25] Sheridan C (2019). Death by inflammation: drug makers chase the master controller. Nat. Biotechnol..

[26] Harris PA (2019). Discovery and lead-optimization of 4,5-dihydropyrazoles as mono-kinase selective, orally bioavailable and efficacious inhibitors of receptor interacting protein 1 (RIP1) kinase. J. Med. Chem..

[27] Harris PA (2017). Discovery of a first-in-class receptor interacting protein 1 (RIP1) kinase specific clinical candidate (GSK2982772) for the treatment of inflammatory diseases. J. Med. Chem..

[28] Hamilton GL (2019). Potent and selective inhibitors of receptor-interacting protein kinase 1 that lack an aromatic back pocket group. Bioorg. Med. Chem. Lett..

[29] Menon MB (2017). p38(MAPK)/MK2-dependent phosphorylation controls cytotoxic RIPK1 signalling in inflammation and infection. Nat. Cell Biol..

[30] Jaco I (2017). MK2 phosphorylates RIPK1 to prevent TNF-induced cell death. Mol. Cell.

[31] Geng J (2017). Regulation of RIPK1 activation by TAK1-mediated phosphorylation dictates apoptosis and necroptosis. Nat. Commun..

[32] Dondelinger Y (2019). Serine 25 phosphorylation inhibits RIPK1 kinase-dependent cell death in models of infection and inflammation. Nat. Commun..

[33] Lafont E (2018). TBK1 and IKKepsilon prevent TNF-induced cell death by RIPK1 phosphorylation. Nat. Cell Biol..

[34] Dondelinger Y (2015). NF-kappaB-independent role of IKKalpha/IKKbeta in preventing RIPK1 kinase-dependent apoptotic and necroptotic cell death during TNF signaling. Mol. Cell.

[35] Dondelinger Y (2017). MK2 phosphorylation of RIPK1 regulates TNF-mediated cell death. Nat. Cell Biol..

[36] Wang X (2014). RNA viruses promote activation of the NLRP3 inflammasome through a RIP1-RIP3-DRP1 signaling pathway. Nat. Immunol..

[37] Degterev A (2008). Identification of RIP1 kinase as a specific cellular target of necrostatins. Nat. Chem. Biol..

[38] Zhang Y (2017). RIP1 autophosphorylation is promoted by mitochondrial ROS and is essential for RIP3 recruitment into necrosome. Nat. Commun..

[39] Wegner KW, Saleh D, Degterev A (2017). Complex pathologic roles of RIPK1 and RIPK3: moving beyond necroptosis. Trends Pharm. Sci..

[40] McQuade T, Cho Y, Chan FK (2013). Positive and negative phosphorylation regulates RIP1- and RIP3-induced programmed necrosis. Biochem. J..

[41] Patel S (2020). RIP1 inhibition blocks inflammatory diseases but not tumor growth or metastases. Cell Death Differ..

[42] Varfolomeev E, Vucic D (2018). Intracellular regulation of TNF activity in health and disease. Cytokine.

[43] Polykratis A (2014). Cutting edge: RIPK1 Kinase inactive mice are viable and protected from TNF-induced necroptosis in vivo. J. Immunol..

[44] Wang L, Du F, Wang X (2008). TNF-alpha induces two distinct caspase-8 activation pathways. Cell.

[45] Dondelinger Y (2014). MLKL compromises plasma membrane integrity by binding to phosphatidylinositol phosphates. Cell Rep..

[46] Cai Z (2014). Plasma membrane translocation of trimerized MLKL protein is required for TNF-induced necroptosis. Nat. Cell Biol..

[47] Nenci A (2007). Epithelial NEMO links innate immunity to chronic intestinal inflammation. Nature.

[48] Luedde T (2007). Deletion of NEMO/IKKgamma in liver parenchymal cells causes steatohepatitis and hepatocellular carcinoma. Cancer Cell.

[49] Ehlken H (2014). Death receptor-independent FADD signalling triggers hepatitis and hepatocellular carcinoma in mice with liver parenchymal cell-specific NEMO knockout. Cell Death Differ..

[50] Gijbels MJ (1996). Pathogenesis of skin lesions in mice with chronic proliferative dermatitis (cpdm/cpdm). Am. J. Pathol..

[51] Seymour RE (2007). Spontaneous mutations in the mouse Sharpin gene result in multiorgan inflammation, immune system dysregulation and dermatitis. Genes Immun..

[52] Kumari S., et al. Sharpin prevents skin inflammation by inhibiting TNFR1-induced keratinocyte apoptosis. *Elife* **3**, (2014). 10.7554/eLife.03422 PMC4225491 25443631

[53] Rickard J. A., et al. TNFR1-dependent cell death drives inflammation in Sharpin-deficient mice. *Elife* **3**, (2014). 10.7554/eLife.03464 PMC4270099 25443632

[54] Schmidt-Supprian M (2000). NEMO/IKK gamma-deficient mice model incontinentia pigmenti. Mol. Cell.

[55] Madison BB (2002). Cis elements of the villin gene control expression in restricted domains of the vertical (crypt) and horizontal (duodenum, cecum) axes of the intestine. J. Biol. Chem..

[56] Kellendonk C, Opherk C, Anlag K, Schutz G, Tronche F (2000). Hepatocyte-specific expression of Cre recombinase. Genesis.

[57] Adolph TE (2013). Paneth cells as a site of origin for intestinal inflammation. Nature.

[58] Newton K (2014). Activity of protein kinase RIPK3 determines whether cells die by necroptosis or apoptosis. Science.

[59] Nolte H, MacVicar TD, Tellkamp F, Kruger M (2018). Instant clue: a software suite for interactive data visualization and analysis. Sci. Rep..

[60] Cox J (2011). Andromeda: a peptide search engine integrated into the MaxQuant environment. J. Proteome. Res..

[61] Tyanova S (2016). The Perseus computational platform for comprehensive analysis of (prote)omics data. Nat. Methods.

[62] Edgar R, Domrachev M, Lash AE (2002). Gene Expression Omnibus: NCBI gene expression and hybridization array data repository. Nucleic Acids Res..

[63] Perez-Riverol Y (2019). The PRIDE database and related tools and resources in 2019: improving support for quantification data. Nucleic Acids Res..

