# Supplementary material for: Autophosphorylation at serine 166 regulates RIP kinase 1-mediated cell death and inflammation
Source: Nat Commun. 2020 Apr 8;11:1747. doi: 10.1038/s41467-020-15466-8 (PMC7142081; doi:10.1038/s41467-020-15466-8)
Supplement: Supplementary file 1 — Supplementary Information [file 41467_2020_15466_MOESM1_ESM.pdf]

Autophosphorylation at serine 166 regulates RIP kinase 1-mediated cell death and inflammation

Laurien *et al.*

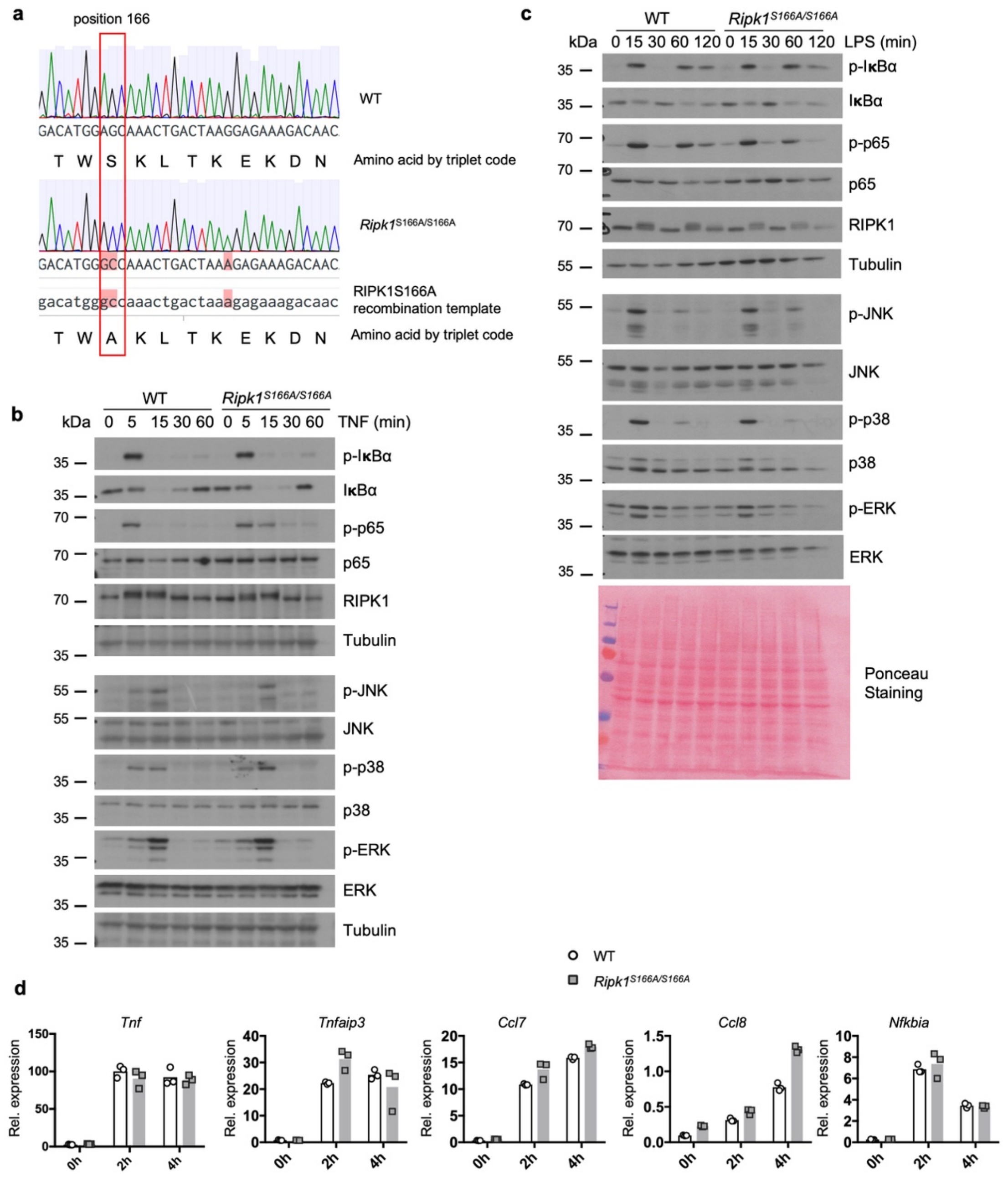

Supplementary Figure 1: RIPK1S166A mutation does not affect TNF- and LPS-induced NF-κB and MAPK activation. a, DNA sequence alignment (5'-3') of the region surrounding the mutation site in WT and *Ripk1*$^{S166A/S166A}$ mice. Amino acids by triplet code are shown. **b,** BMDMs from mice of the indicated genotypes were treated with TNF (10 ng ml^{-1}) for 0, 5, 15, 30 and 60 min. Cell lysates were analyzed by immunoblot with the indicated antibodies. One representative out of three independent experiments is shown. **c,** BMDMs from mice of the indicated genotypes were treated with LPS (100 ng ml^{-1}) for 0, 15, 30, 60 and 120 min. Cell lysates were analyzed by immunoblot with the indicated antibodies. Data represent one experiment. **d,** Graphs depicting relative mRNA expression (rel. expression) of the indicated genes from BMDMs treated with LPS for 0, 2 or 4 hours, measured by qRT-PCR. Bar graphs show the mean of three technical replicates. Individual values of technical replicates are shown as single data points.

Fig 1a

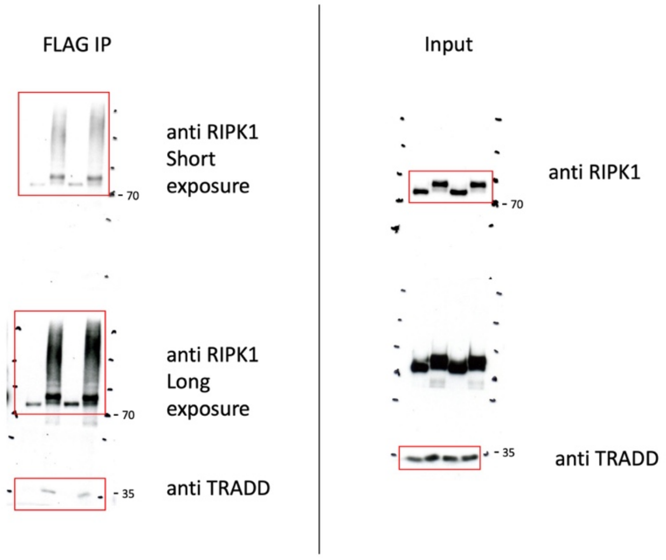

Supplementary Figure 2: Source data for immunoblots. Uncropped scans of all the immunoblots presented in the manuscript

Supplementary Figure 2 (continued)

Figure S1b

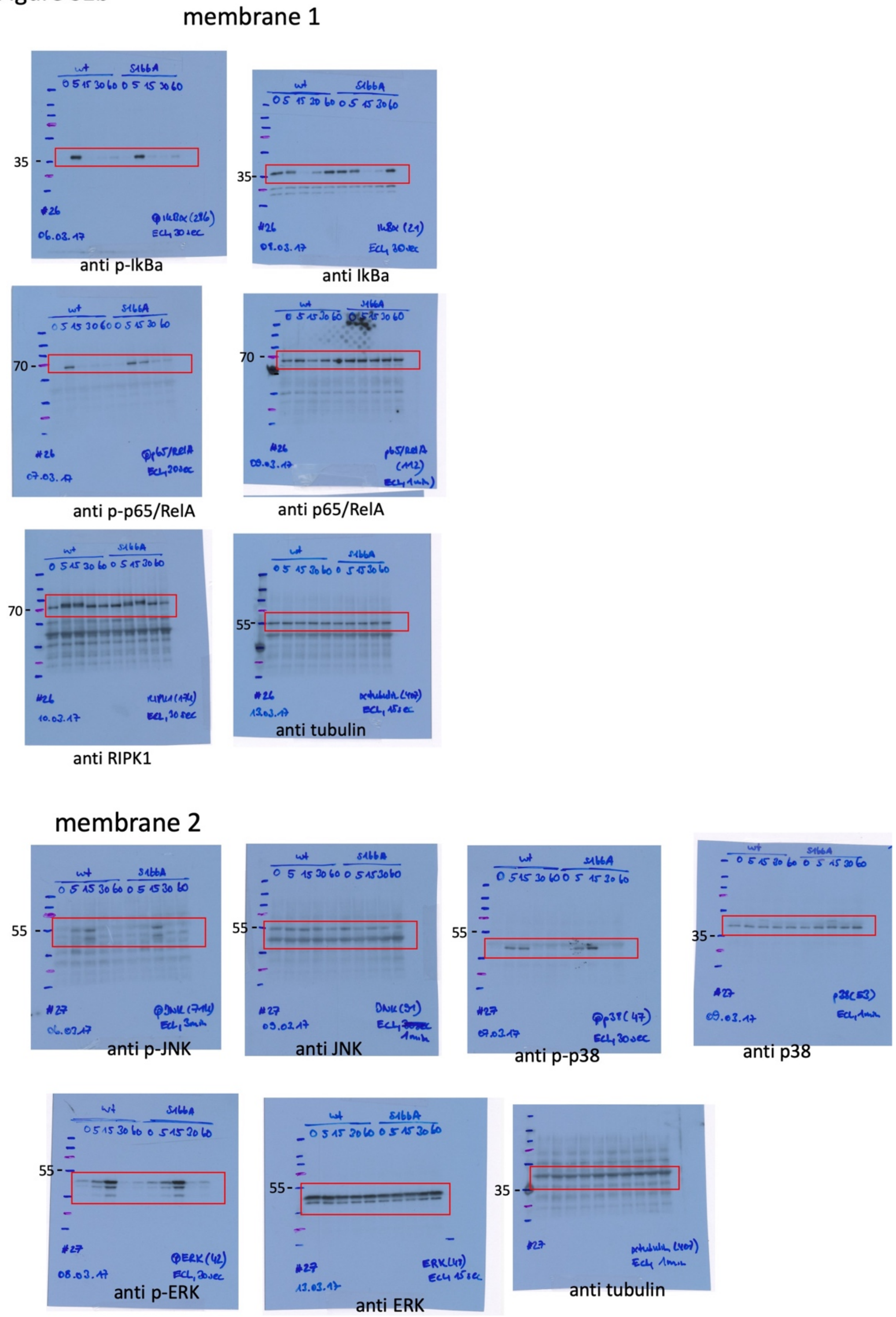

Supplementary Figure 2 (continued)

Figure S1c

membrane 1

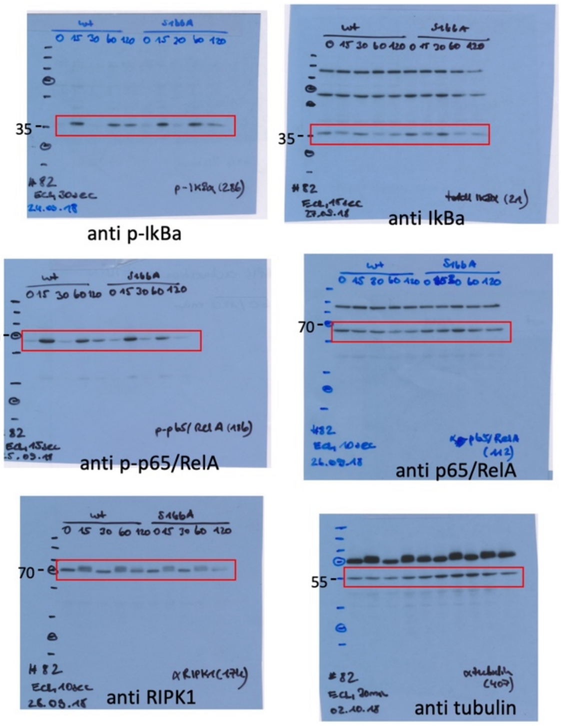

membrane 2

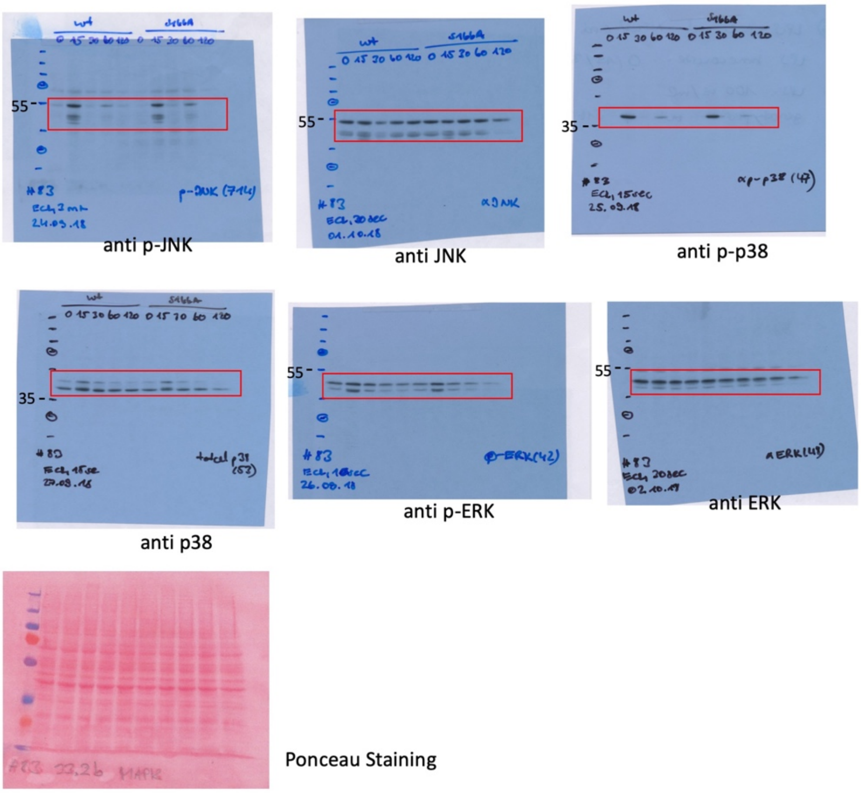

Supplementary Figure 2 (continued)

Figure 2a

Input

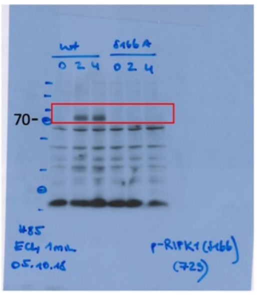

anti p-RIPK1 (S166)

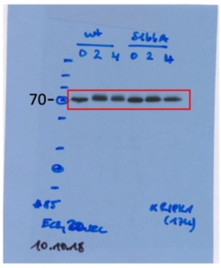

anti RIPK1

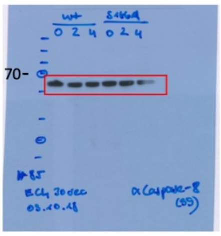

anti Caspase8

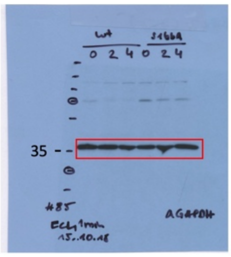

anti GAPDH

IP

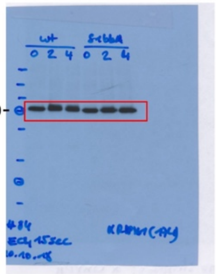

anti RIPK1

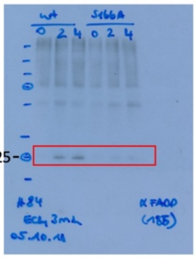

anti FADD

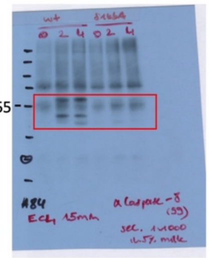

anti Caspase-8

Figure 2b

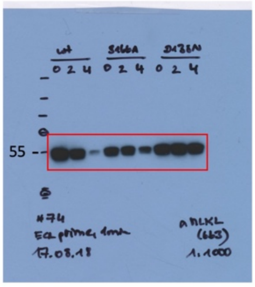

anti MLKL,
short exposure

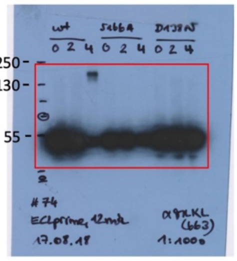

anti MLKL,
Long exposure

Supplementary Figure 2 (continued)

Fig 2c

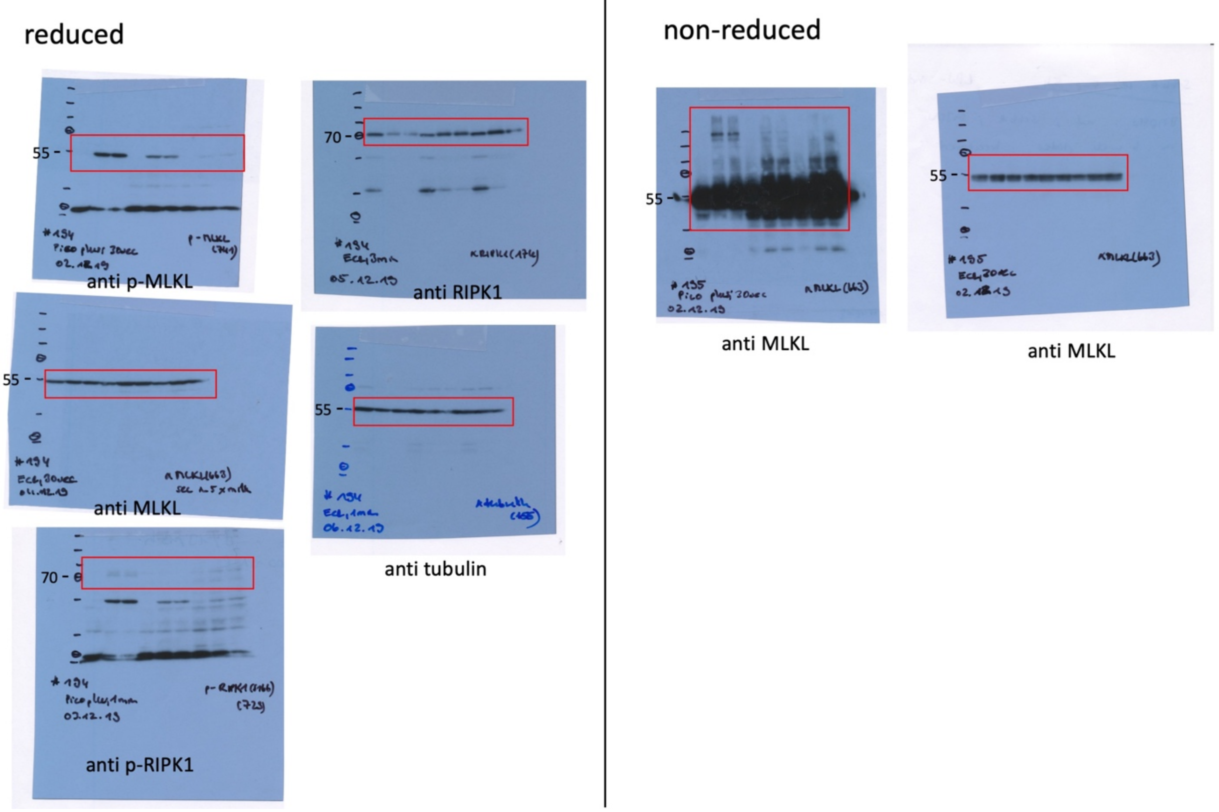

Fig 2d

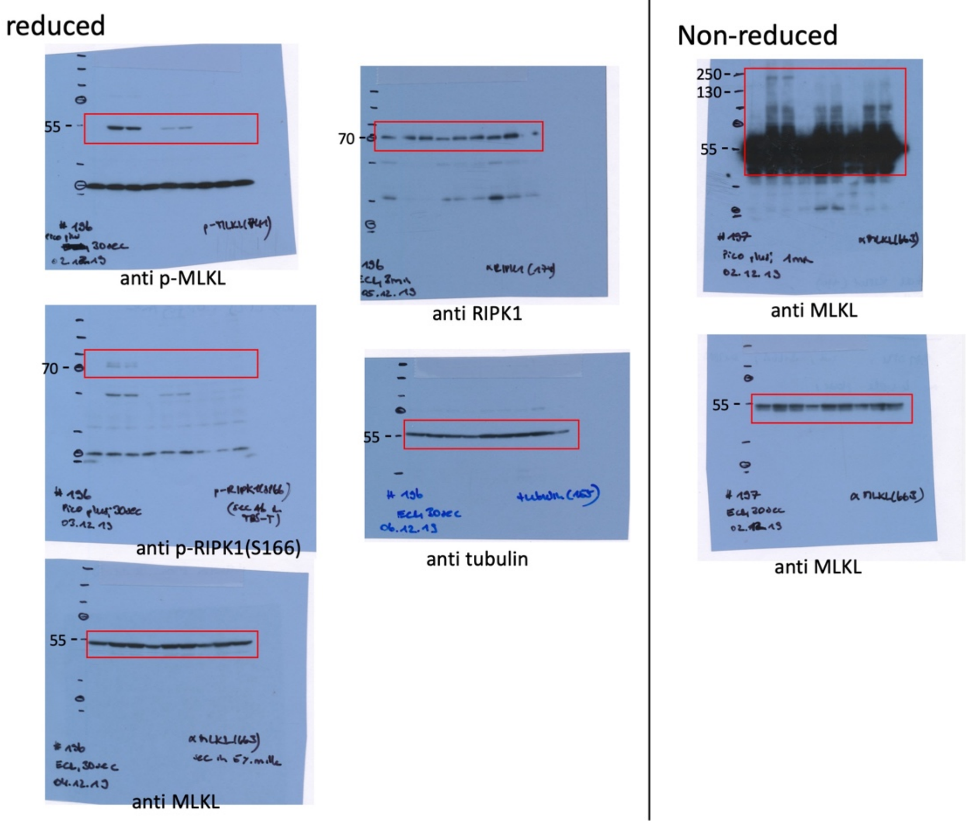

Supplementary Figure 2 (continued)

Fig. 7 b

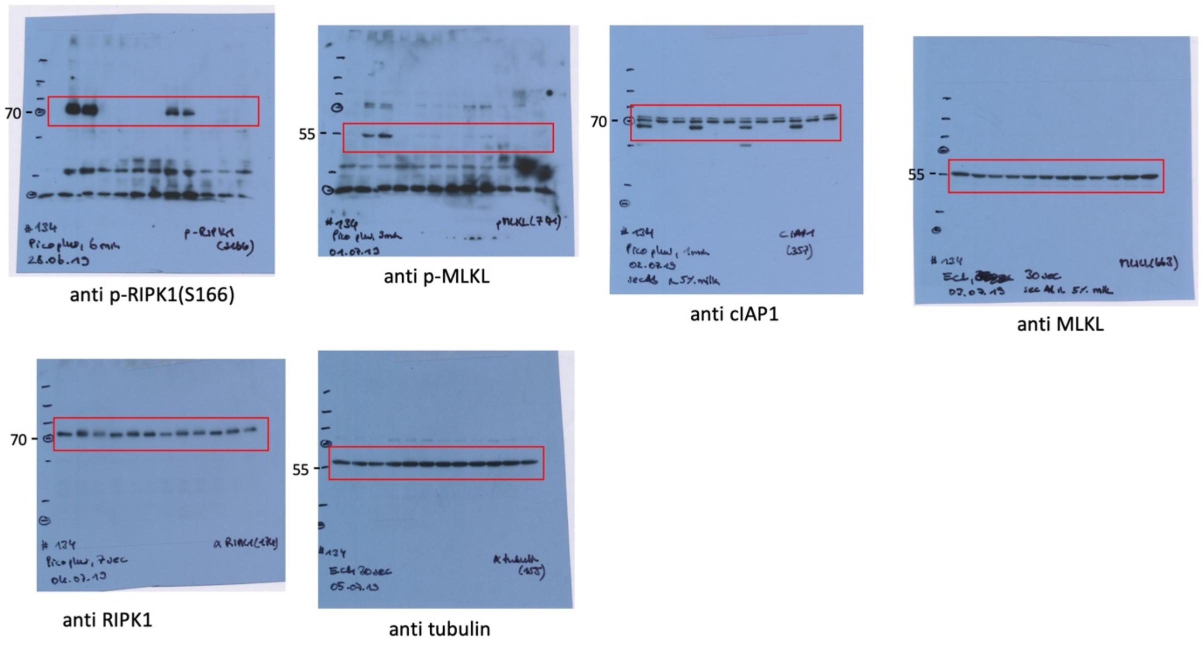

Fig. 7 c

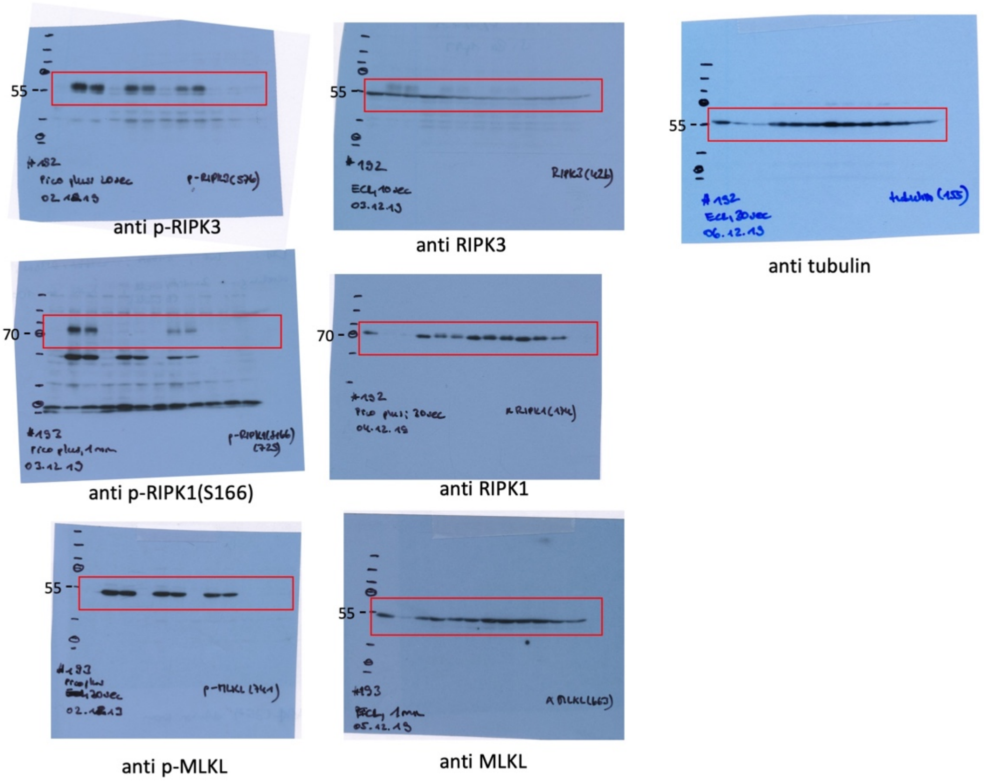

Supplementary Figure 2 (continued)

Fig. 7 e

FADD-IP

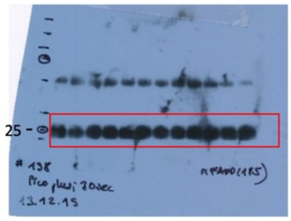

anti FADD

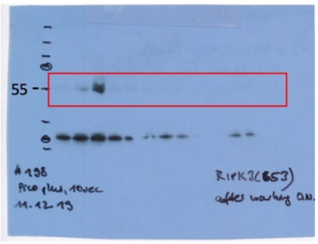

anti RIPK3

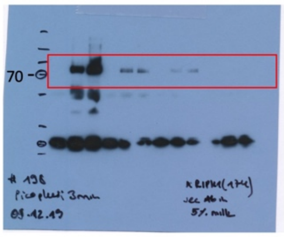

anti RIPK1

Input

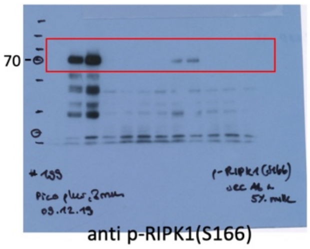

anti p-RIPK1(S166)

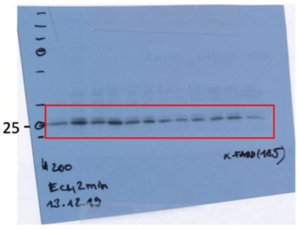

anti FADD

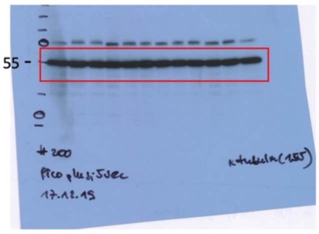

anti Tubulin

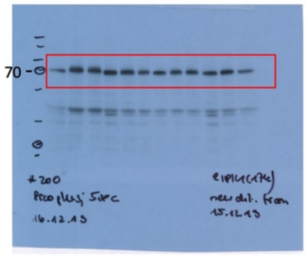

anti RIPK1

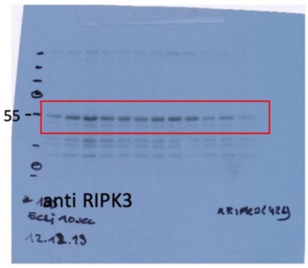

anti RIPK3

Supplementary Figure 2 (continued)

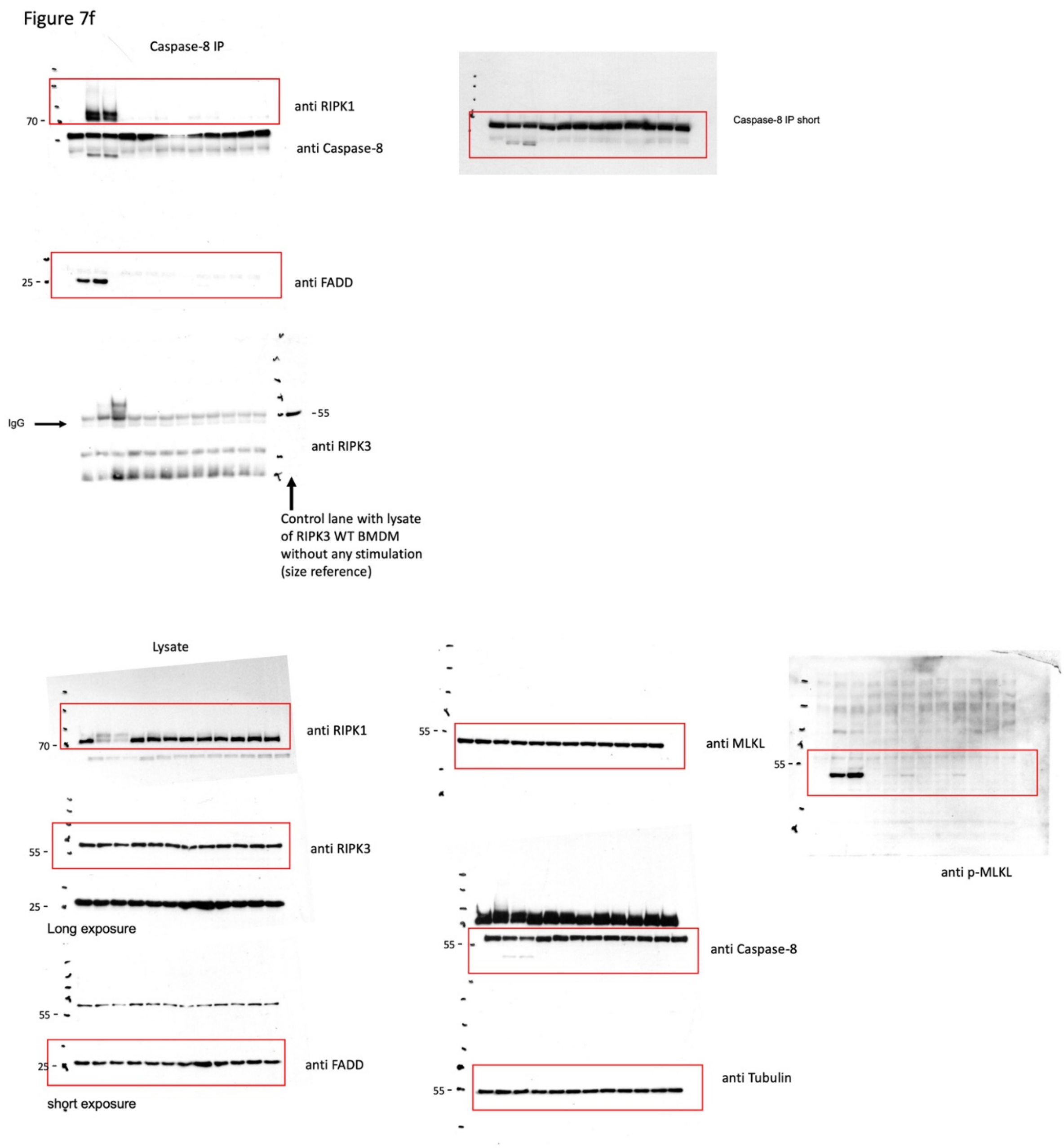

Supplementary Figure 2 (continued)

Validation of anti-RIPK1 serum

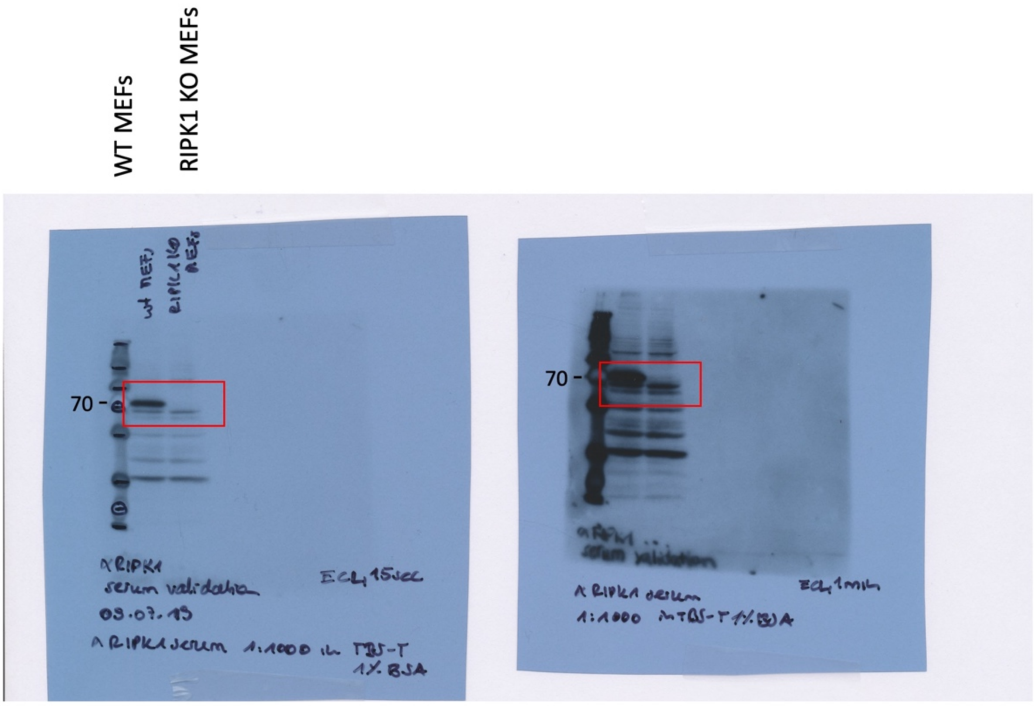

anti RIPK1 homemade serum, Short exposure

anti RIPK1 homemade serum Long exposure
