# Supplementary material for: Autophosphorylation at serine 166 regulates RIP kinase 1-mediated cell death and inflammation
Source: Nat Commun. 2020 Apr 8;11:1747. doi: 10.1038/s41467-020-15466-8 (PMC7142081; doi:10.1038/s41467-020-15466-8)
Supplement: Supplementary file 2 — Reporting Summary [file 41467_2020_15466_MOESM2_ESM.pdf]

nature research

Corresponding author(s): Manolis Pasparakis

Reporting Summary

Nature Research wishes to improve the reproducibility of the work that we publish. This form provides structure for consistency and transparency in reporting. For further information on Nature Research policies, see Authors & Referees and the Editorial Policy Checklist.

Statistical parameters

When statistical analyses are reported, confirm that the following items are present in the relevant location (e.g. figure legend, table legend, main text, or Methods section).

n/a	Confirmed	
☐	☒	The exact sample size (*n*) for each experimental group/condition, given as a discrete number and unit of measurement
☐	☒	An indication of whether measurements were taken from distinct samples or whether the same sample was measured repeatedly
☐	☒	The statistical test(s) used AND whether they are one- or two-sided<br>*Only common tests should be described solely by name; describe more complex techniques in the Methods section.*
☒	☐	A description of all covariates tested
☐	☒	A description of any assumptions or corrections, such as tests of normality and adjustment for multiple comparisons
☐	☒	A full description of the statistics including central tendency (e.g. means) or other basic estimates (e.g. regression coefficient) AND variation (e.g. standard deviation) or associated estimates of uncertainty (e.g. confidence intervals)
☐	☒	For null hypothesis testing, the test statistic (e.g. *F*, *t*, *r*) with confidence intervals, effect sizes, degrees of freedom and *P* value noted<br>*Give P values as exact values whenever suitable.*
☒	☐	For Bayesian analysis, information on the choice of priors and Markov chain Monte Carlo settings
☒	☐	For hierarchical and complex designs, identification of the appropriate level for tests and full reporting of outcomes
☒	☐	Estimates of effect sizes (e.g. Cohen's *d*, Pearson's *r*), indicating how they were calculated
☐	☒	Clearly defined error bars<br>*State explicitly what error bars represent (e.g. SD, SE, CI)*

Our web collection on statistics for biologists may be useful.

Software and code

Policy information about availability of computer code

Data collection	No software was used for data collection.
Data analysis	Statistical analysis was performed with GraphPad Prism V6. DNA sequence alignment was performed using Benchling.com. Quantification of live and dead cell numbers was performed using the built-in IncuCyte software (Version S3). Heat map of microarray data was built using InstantClue software (Version 0.4.9). Raw files resulting from MS/MS scans were processed using Max Quant software (Version 1.5.3.8). Phosphosite intensities were logarithmized and normalized using Perseus software (Version 1.5.5.3). Array-Quality Control for Clariom-S-mouse microarray was done using the Affymetrix Expression Console (Version 1.4) or Transcriptome Analysis Console (TAC, Version 4.0) Software.

For manuscripts utilizing custom algorithms or software that are central to the research but not yet described in published literature, software must be made available to editors/reviewers upon request. We strongly encourage code deposition in a community repository (e.g. GitHub). See the Nature Research guidelines for submitting code & software for further information.

Data

Policy information about availability of data

All manuscripts must include a data availability statement. This statement should provide the following information, where applicable:

- Accession codes, unique identifiers, or web links for publicly available datasets
- A list of figures that have associated raw data
- A description of any restrictions on data availability

The microarray data discussed in this publication have been deposited in NCBI's Gene Expression Omnibus 60and are accessible through GEO Series accession number GSE 131855 (https://ncbi.nih.gov/geo/query/acc.cgi?acc=GSE131855). The mass spectrometry proteomics data have been deposited to the ProteomeXchange Consortium via the PRIDE 61 partner repository with the dataset identifier PXD014097. The source data underlying Figs 1b-i, 3b–d, 4a, e-f, 5a, d, 6a, b, and 7d and Supplementary Fig. 1d are provided as a Source Data file. Uncropped images of immunoblots presented in the figures are included in "Supplementary Fig. 2".

Field-specific reporting

Please select the best fit for your research. If you are not sure, read the appropriate sections before making your selection.

☒ Life sciences ☐ Behavioural & social sciences ☐ Ecological, evolutionary & environmental sciences

For a reference copy of the document with all sections, see nature.com/authors/policies/ReportingSummary-flat.pdf

Life sciences study design

All studies must disclose on these points even when the disclosure is negative.

Sample size	Based on our previous experience with disease mouse models used in this study (Vlantis et al. 2016 (15), Kondylis et al 2015 (14), Kumari et al. 2014 (52)) we aimed to analyze at least 5 animals per group to evaluate the effect of the RIPK1S166A mutation on cell death and inflammation in different tissues between different groups of genetically modified mice . Based on previous experience from similar studies (Lin et al. 2016 (9), Polykratis et al. 2014 (43), in vitro experiments with cultured cells were performed at least 2-3 times (biological replicates) to confirm reproducibility.
Data exclusions	No data was excluded from the analysis
Replication	For our in vivo studies we analyzed a sufficient number of animals (at least 5 mice per group, less in rare exceptions) to ensure that the results obtained are consistent. For in vitro studies in primary cells we replicated all experiments in at least 2-3 independently isolated primary cell batches from individual mice (biological replicates). All attempts of replication were succesfull.
Randomization	No specific method of randomization had been used to select animals. We compared groups of mice with different genotypes to assess the effect of specific genetic mutations in the phenotype. Group allocation was thus determined by the genotype of the mice. We did not specifically control for covariates in this study.
Blinding	Histological scoring of colon tissue sections was performed blindly. Except for histological analysis no blinding to group allocation was done during data collection and/or analysis as this was not practical for experiments performed by a single investigator.

Reporting for specific materials, systems and methods

Materials & experimental systems

n/a	Involved in the study	
☒	☐	Unique biological materials
☐	☒	Antibodies
☒	☐	Eukaryotic cell lines
☒	☐	Palaeontology
☐	☒	Animals and other organisms
☒	☐	Human research participants

Methods

n/a	Involved in the study	
☒	☐	ChIP-seq
☒	☐	Flow cytometry
☒	☐	MRI-based neuroimaging

Antibodies

Antibodies used	Antibodies against the following proteins were used for Immunoblotting/Immunohistochemistry:

1) monoclonal rabbit anti-p-IκBα, Cat. No. 2859, Cell Signaling Technology; dilution 1:1000 for WB, Lot. No. 17
2) polyclonal rabbit anti-IκBα, Cat. No. sc-371, Santa Cruz Biotechnology; dilution 1:1000 for WB, Lot. No. K1315
3) monoclonal rabbit anti-p-p65, Cat. No. 3033, Cell Signaling Technology; dilution 1:1000 for WB, Lot. No. 16
4) polyclonal rabbit anti-p65, Cat . No sc-372, Santa Cruz Biotechnology; dilution 1:1000 for WB, Lot. No. K0415
5) polyclonal rabbit anti-p-SAPK/JNK (T183/T185), Cat. No. 4668, Cell Signaling Technology; dilution 1:1000 for WB, Lot. No. 15
6) polyclonal rabbit anti-SAPK/JNK, Cat. No. 9252, Cell Signaling Technology; dilution 1:1000 for WB, Lot. No. 17
7) polyclonal rabbit anti-p-p38, Cat. No. 9211, Cell Signaling Technology; dilution 1:1000 for WB, Lot. No. 25
8) polyclonal rabbit anti-p38, Cat. No. 9212, Cell Signaling Technology; dilution 1:1000 for WB, Lot. No. 17
9) polyclonal rabbit anti-p-p44/42 MAPK (ERK1/2), Cat. No. 9191, Cell Signaling Technology; dilution 1:1000 for WB, Lot. No. 29
10) polyclonal rabbit anti-p44/42 MAPK (ERK1/2), Cat. No. 9102, Cell Signaling Technology; dilution 1:1000 for WB, Lot. No. 27
11) monoclonal mouse anti-RIPK1, Cat. No. 610459, BD Biosciences; dilution 1:1000 for WB, Lot. No. 9038922
12) custom-made polyclonal rabbit anti-RIPK1 serum
13) monoclonal rabbit anti RIPK1, Cat. No. 3493, Cell Signaling Technology; dilution 1:1000 for WB
14) polyclonal rabbit anti-p-RIPK1(S166), Cat. No. 31122, Cell Signaling Technology; dilution 1:1000 for WB, Lot No. 3
15) polyclonal rabbit anti-p-RIPK3, Cat. No. 57220, Cell Signaling Technology, dilution 1:1000 for WB, Lot No. 1
16) polyclonal rabbit anti-RIPK3, Cat. No. ADI-905-242-100, Enzo Life Sciences, dilution 1:1000 for WB, Lot No. 06081805
17) polyclonal rat anti RIPK3, clone 1G6.14 (Ref. 58), Genentech Inc., dilution 1:1000 for WB
18) polyclonal rabbit anti RIPK3, Cat. No. PA5-19956, Thermo Fisher Scientific, dilution 1:1000 for WB
19) monoclonal rabbit anti-p-MLKL (S345), Cat. No. D6E3G, Cell Signaling Technology; dilution 1:1000 for WB, Lot No. 2
20) monoclonal rabbit anti p-MLKL, Cat. No. ab196436 clone EPR9515(2), Abcam, dilution 1:1000 in WB
21) monoclonal rat anti-MLKL, Cat. No. MABC604, Millipore; dilution 1:1000 for WB, Lot. No 3256617
22) monoclonal rat anti-Caspase 8, Cat. No. ALX-804-447, Alexis; dilution 1:1000 for WB, Lot No. 08271911
23)monoclonal mouse anti-FADD, Cat. No. 05-486, upstate; dilution 1:1000 for WB
24)polyclonal goat anti-FADD, Cat. No. sc-6036, Santa Cruz Biotechnology; for IP, Lot No. H2615
25) monoclonal mouse anti FADD, Cat. No. ADI-AAM-212-E, Enzo Life Sciences, dilution 1:1000 for WB 25) polyclonal rabbit anti TRADD, Cat. No. AHP2533, Bio-Rad Laboratories, dilution 1:1000 for WB
26) monoclonal mouse anti-GAPDH, Cat. No. NB300-221, Novus Biologicals, dilution 1:1000 for WB, Lot No. 082219
27) monoclonal mouse anti-Tubulin, Cat. No. T6074, Sigma-Aldrich; dilution 1:1000 for WB, Lot No. 118M4779
28) polyclonal rabbit anti Tubulin, Cat. No. ab21058, Abcam, dilution 1:15 000 for WB
29) sheep anti-mouse IgG horseradish peroxidase (HRP)-linked antibody, Cat. No. NA931, GE Healthcare, dilution 1:10 000 for WB, Lot No.
30) sheep anti-rabbit IgG conjugated to HRP antibody, Cat. No. NA934V, GE Healthcare, dilution 1: 10 000 for WB
31) goat anti-rat IgG HRP-linked antibody, Cat. No. 112-035-003, Jackson Immuno Research, dilution 1: 10 000 for WB
32) polyclonal rabbit anti-cleaved Caspase3, Cat. No. 9661, Cell Signaling Technology; Lot. No. 43
33) monoclonal rat anti-Ki67, Cat. No. M724901, DAKO, dilution for IHC 1:1000, Lot No.
34) monoclonal mouse anti-SMA, Cat. No. A2547, Sigma-Aldrich, dilution for IHC 1:1000, Lot No.
35) monoclonal rat anti-F4/80, Cat. No. MCA497, clone A3-1, AbD Serotec, dilution for IHC 1:100,
36) polyclonal rabbit anti-Keratin 14, Cat. No. MS-115, Neomarkers, dilution for IHC 1:400,
37) polyclonal rabbit anti-Keratin 6, Cat. No. PRB-169P, Covance, dilution 1:600 for IHC
38) polyclonal rabbit anti-Keratin 10, Cat. No. PRB-159P, Covance, dilution 1:300 for IHC
39) anti mouse Alexa 488- fluorescence-conjugated secondary Ab, Cat. No. A1101, Molecular Probes, dilution 1:800 for IHC
40) anti rabbit Alexa 549- fluorescence-conjugated secondary Ab, Cat. No. A11012, Molecular Probes, dilution 1:800 for IHC

Validation

Validation data for all commercial antibodies are available on vendor websites.
Custom-made anti RIPK1 antibody is validated for WB in this manuscipt in RIPK1 KO MEFs.

Animals and other organisms

Policy information about studies involving animals; ARRIVE guidelines recommended for reporting animal research

Laboratory animals

Ripk1S166A/S166A mice were generated in cooperation with the CECAD Transgenic Core Facility. Cas9 mRNA (TriLink) together with the 120 bp ssDNA repair oligo (IDT) and the short guide RNA(s) (sgRNA) were microinjected into the pronucleus of fertilized oocytes obtained from C57BL/6 mice. The sgRNAs targeted a site adjacent to position 166 of the murine Ripk1 gene (5’gacatggagcaaactgacta3’). Founder mice were then backcrossed to C57BL/6 mice.
Ripk1D138N/D138N mice (Polykratis et al., 43), Nemo FL (Schmidt-Supprian et al.,54), Villin-Cre (Madison et al., 55), AlfpCre (Kellendonk et al., 56) and Sharpin cpdm/cpdm (Gijbels et al., 1996, 50) mice have been described before. All in vivo experiments were performed with littermate mice.

Wild animals

The study did not involve wild animals

Field-collected samples

The study did not involve samples collected from the field.
